# Computing Rate–Distortion Functions of Continuous Memoryless Sources via Discrete Algorithms: An Integrated Scheme with Convergence Guarantee and Algorithmic Acceleration

**DOI:** 10.3390/e28030280

**Published:** 2026-03-01

**Authors:** Lingyi Chen, Haoran Tang, Hao Wu, Huihui Wu, Shitong Wu, Wenyi Zhang

**Affiliations:** 1 Department of Mathematical Sciences, Tsinghua University, Beijing 100084, China; cly23@mails.tsinghua.edu.cn (L.C.); thr22@mails.tsinghua.edu.cn (H.T.); hwu@tsinghua.edu.cn (H.W.); wushitong1234@163.com (S.W.); 2 Ningbo Institute of Digital Twin, Ningbo 315200, China; huihui.wu@ieee.org; 3 Department of Electronic Engineering and Information Science, University of Science and Technology of China, Hefei 230027, China

**Keywords:** rate–distortion function, continuous memoryless sources, Blahut–Arimoto algorithm, convergence analysis, fast algorithms

## Abstract

Numerical computation of the rate–distortion (RD) function is a key problem in RD theory. Thus far, efficient algorithms have been well studied for discrete sources, but for continuous sources, there is still lack of a rigorously developed solution. In this article, an integrated approach is conducted that bridges RD problems of continuous memoryless sources and discrete numerical algorithms. First, we analyze and establish the theoretical convergence guarantee when progressively approaching the continuous RD problem via a sequence of discrete problems. Next, discrete algorithms including the Blahut–Arimoto (BA) and constrained BA algorithms are reviewed, and estimates of their required amount of arithmetic operations to attain ε-accuracy in solving the continuous RD problem are derived. Finally, acceleration techniques that exploit structures of special distortion measures (i.e., squared-error and absolute-error) are developed.

## 1. Introduction

Rate–distortion (RD) theory traces back to Shannon’s seminal work on lossy source coding, where the RD function was introduced to characterize the fundamental trade-off between rate and distortion for representing a source subject to a fidelity criterion [1]. RD theory has been instrumental to the development of signal quantization and compression [2], and has long-lasting impacts on standards for media compression [3,4,5]. See [6] for a systematic treatment of standard and classical aspects of RD theory, [7] for a survey on the development of RD theory, [8] for introduction on RD theory in distributed settings, and [9] for recent progresses on the interplay between RD theory and machine learning.

Evaluating the RD function is a key problem in rate–distortion theory, and due to the lack of closed-form expressions in general settings, numerical algorithms are usually necessary. Thus far, efficient algorithms have been well studied for discrete sources, including the famous Blahut–Arimoto (BA) algorithm [10,11] and several others such as the constrained BA (CBA) algorithm [12] and expectation–maximization (EM)-type algorithms utilizing Bregman projections or mirror descent [13]. But for continuous sources, there is still lack of a rigorously developed solution. A commonly adopted approach is first discretizing the source and reproduction spaces and then solving the resulting discrete RD problem. Do solutions of the discretized problems provably converge to those of the original continuous problem? This basic question has remained unanswered to the best of our knowledge. It should be noted that, due to the nonlinear optimization formulation of the RD problem, naive discretization does not necessarily yield a convergent sequence of solutions towards those of the original continuous problem [14].

This article conducts an integrated approach for the computation of RD functions, bridging continuous RD problems and discrete numerical algorithms. Specifically, we (i) for continuous RD problems, construct suitable discretizations and prove that their solutions converge to those of the original continuous problem; (ii) review, based on [12], estimates of the required amount of arithmetic operations when applying the BA and CBA algorithms for attaining ε-accuracy within the solution of the continuous RD problem; and (iii) design acceleration techniques that substantially reduce time and memory costs for squared-error and absolute-error distortion measures. A preliminary result pertaining to (i) was first presented in part at the IEEE International Symposium on Information Theory (ISIT) in 2024 [15], while here we present the complete work with proofs.

In our approach, we analyze a suitable dual form of the RD function, constructing a sequence of finite-dimensional spaces that progressively approximate the infinite-dimensional space of reproduction distributions. The convergence of discrete problem solutions to the continuous one is established by using standard tools in mathematical analysis [16]. The proof is algorithm-agnostic and does not depend on specific numerical algorithms used. In an alternative approach, mapping-based methods jointly optimize locations and masses of the discrete reproduction distribution [17], but the explicit dependence on locations makes the optimization problem nonconvex and typically lacks uniform performance guarantees [18]. By avoiding optimization over locations and instead working in the limit of asymptotically vanishing discretization step size, our approach provably guarantees convergence of both objective values and solution sequences. In another work [19], the RD function under a fixed output alphabet size constraint is evaluated, and corresponding encoding schemes are proposed.

With convergence guarantees, computing the continuous RD function can be effectively reduced to solving the discretized RD problems with numerical algorithms, i.e., the BA or CBA algorithm. To assess the efficiency of this approach, we review the arithmetic complexity required to attain an ε-accurate solution. According to the analysis in [12], for the CBA algorithm, this complexity is $O\left(\frac{\left|\log\varepsilon \right|}{\varepsilon^{d+1}}\left(1+\log|\log\varepsilon |\right)\right)$ multiplied by the number of discretization nodes, where d=dim(Y) is the reproduction dimension. Since achieving ε-accuracy in *d*-dimensional integration necessitates a grid with $O(1/\varepsilon^{d})$ nodes, the overall complexity becomes $O\left(\frac{\left|\log\varepsilon \right|}{\varepsilon^{2d+1}}(1+\log|\log\varepsilon |)\right)$. Clearly, this leads to substantial computational complexity.

To mitigate the aforementioned substantial computational complexity, we design fast algorithms by exploiting the structure of uniform discretization. Specifically, for squared-error distortion, we leverage the Fast Fourier Transform (FFT) to accelerate the iterated convolutions in the CBA updates, achieving an acceleration factor of $\left|\log\varepsilon \right|/\varepsilon^{d}$. For absolute-error distortion, we devise a fast algorithm based on dynamic programming ideas, inspired by [20], which achieves an acceleration factor of $1/\varepsilon^{d}$.

The remaining part of this article is organized as follows. Section 2 develops the continuous RD framework, formulates its discretizations, and establishes the convergence of the associated discrete schemes. Section 3 reviews the BA and CBA algorithms, and provides estimates on their required amount of arithmetic operations for attaining ε-accuracy within the solution of the continuous RD problem. Section 4 further develops acceleration techniques, including FFT-based acceleration for squared-error distortion and nearly linear time acceleration for absolute-error distortion, with reduced time and memory costs. Section 5 concludes this article.

## 2. Discretization of Continuous RD Problems and Convergence Analysis

In this section, we address the discretization of continuous RD problems and analyze the convergence of the resulting discrete schemes.

### 2.1. Continuous RD Problems and Their Discretizations

Consider a continuous memoryless source X∈X with reproduction Y∈Y, where X,Y are finite-dimensional Euclidean spaces. Denote the dimension of Y as *d*. Let *p* and r denote the probability distributions of *X* and *Y*, respectively, and let $\rho :X\times Y\to R_{\geq 0}$ be a distortion measure. For an average distortion threshold D≥0, the RD function [6,21] is

(1) $$R\left(D\right)\;≜\;\min_{P_{Y|X}}I\left(X;Y\right)\;s.t.\;E_{P_{XY}}\;\left[\rho (X,Y)\right]\leq D,$$

where $P_{XY}=P_{X}P_{Y|X}$. Define F(r,β) as

(2) $$F\left(r,\beta \right)≜-\int \log\;\left[\int \exp\;\left(-\beta \;\rho (x,y)\right)\;dr\left(y\right)\right]\;dp\left(x\right)\;-\;\beta D.$$

A convenient max–min formulation of the RD problem (cf. [22] (Equation (7))) is

(3) $$\max_{\beta \geq 0}\;\min_{r\in W}F\left(r,\beta \right),$$

where W={r:∫dr(y)=1} and *D* denotes the average distortion threshold. Here r is understood as a probability measure on Y equipped with the topology of weak convergence [23], for which it is well-known that for continuous sources under the squared-error distortion measure, the optimal reproduction distribution r is usually discrete [17,24].

In (3), by fixing the multiplier β and defining f(r) as

(4) $$f\left(r\right)≜-\int \log\;\left[\int \exp\;\left(-\beta \;\rho (x,y)\right)\;dr\left(y\right)\right]\;dp\left(x\right),$$

the inner minimization of (3) can be rewritten as (see [17] (Equation (3)):

(5) $$\min_{r\in W}\;f\left(r\right).$$

Since r(y) is defined over the continuum W, it is common to discretize it on a uniform grid of points ${\left\{y_{j}^{n}\right\}}_{j=1}^{n}$ within ${\left[-n^{\frac{1}{2d}},n^{\frac{1}{2d}}\right]}^{d}$. We denote the discretization step size as *h*, i.e., $h=2n^{-\frac{1}{2d}}$, and the interval $\left[y_{j}^{n}-h/2,y_{j}^{n}+h/2\right]$ containing $y_{j}$ as $I_{j}$, noting that these intervals are disjoint. This thus leads to the following discrete form:

(6) $$\min_{r:\sum_{j=1}^{n}r_{j}=1}-\int \log\left[\sum_{j=1}^{n}\exp\left(-\beta \rho \left(x,y_{j}^{n}\right)\right)r_{j}\right]dp\left(x\right).$$

Correspondingly, (3) corresponds to the following discrete form:

(7) $$\max_{\beta \geq 0}\min_{r:\sum_{j=1}^{n}r_{j}=1}\;\;-\;\;\int \;\log\left[\sum_{j=1}^{n}\exp\left(-\beta \rho \left(x,y_{j}^{n}\right)\right)r_{j}\right]dp\left(x\right)-\beta D.$$

The solution of (6) or (7) can be represented as $r^{n}=\sum_{j}r_{j}\delta_{y_{j}^{n}}$, where $\delta_{y_{j}^{n}}$ is the Dirac delta function at $y_{j}^{n}$.

Before proceeding with the subsequent discussion, we clarify some basic notations and terms used throughout the text. ${\left[-M,M\right]}^{d}$ denotes the *d*-fold Cartesian product of [−M,M]; ∥·∥ denotes the $\ell_{\infty }$ norm; $a_{n}\to a$ means convergence in R, while $f_{n}⇉f$ denotes uniform convergence [16], i.e., ∀ε>0, $|f_{n}\left(x\right)-f\left(x\right)|\leq \varepsilon ,\forall x$ when *n* is sufficiently large. For the sequence ${\left\{r^{n}\right\}}_{n=1}^{\infty }$, the limit points [23] are all the points satisfying that every punctured neighborhood contains at least one point in ${\left\{r^{n}\right\}}_{n=1}^{\infty }$, and we denote the set that contains all the limit points of the sequence ${\left\{r^{n}\right\}}_{n=1}^{\infty }$ as $L({\left\{r^{n}\right\}}_{n=1}^{\infty })$. We also write weak convergence as $r^{k}\overset{w}{\to }r$. For brevity, the expression log∫exp(−βρ(x,y))dr(y) will be abbreviated as $H_{r(y)}\left(x\right)$ when no confusion arises. We write $X={\left\{x_{i}\right\}}_{i=1}^{m}$ and $Y={\left\{y_{j}\right\}}_{j=1}^{n}$ for discretized spaces X and Y, respectively.

### 2.2. Convergence of Discrete Schemes

Before proceeding with the analysis, we outline the underlying assumption.

**Assumption** **1.** *The distortion measure ρ and source distribution p satisfy*

$$\begin{array}{cc} \; & \forall \beta >0,\forall \varepsilon >0,\exists \delta >0,\;s.t.\forall x,\forall ∥y_{1}-y_{2}∥<\delta ,\\ \; & |\exp\left(-\beta \rho \left(x,y_{1}\right)\right)-\exp\left(-\beta \rho \left(x,y_{2}\right)\right)|<\varepsilon ,\int \max_{∥y∥\leq A}\rho \left(x,y\right)dp\left(x\right)<\infty ,\forall A>0, \end{array}$$

*and*

$$\begin{array}{cc} \; & |\exp\left(-\beta \rho \left(x,y_{1}\right)\right)\rho \left(x,y_{1}\right)-\exp\left(-\beta \rho \left(x,y_{2}\right)\right)\rho \left(x,y_{2}\right)|<\varepsilon . \end{array}$$

We emphasize that this assumption constitutes general conditions applicable to broad scenarios. With this assumption, we summarize the main results of convergence and convergence rate in the following theorem:

**Theorem** **1.** *The convergence and convergence rate results for discrete problem (7) are as follows:*

*(1)*  *Under the assumption, the solutions $\left(r^{n},\beta^{n}\right)$ to the discrete problem (7) satisfy convergence of the objective values and convergence of the sequence, i.e.,*
$$F\left(r^{n},\beta^{n}\right)\to F^{*},\;and\;L\left({\left\{\left(r^{n},\beta^{n}\right)\right\}}_{n=1}^{\infty }\right)\;are\;solutions\;of\;(3),$$
  where $F^{*}$ is the optimal value of the continuous problem (3).
*(2)*  *When the probability distribution p of X is supported over ${\left[-M,M\right]}^{d}$, the optimal values $F(r^{n},\beta^{n})$ of the discrete problem (7) satisfy the error estimate*
$$|F\left(r^{n},\beta^{n}\right)-F^{*}|\leq Ch,$$
  *where C=C(p,ρ) is a constant independent of n, and $h=2M/n^{\frac{1}{d}}$ is the discretization step size.*

To prove part (1) in Theorem 1, we first consider the case of fixed β, establishing the convergence of the discrete problem (6) to the continuous problem (5).

**Lemma** **1.** *Under the assumption, for any fixed multiplier β>0, the solutions $r^{n}$ to the discrete problem (6) satisfy both value convergence and sequence convergence to (5), i.e.,*

$$f\left(r^{n}\right)\to f^{*},\;and\;L\left({\left\{r^{n}\right\}}_{n=1}^{\infty }\right)\;is\;the\;solution\;set\;of\;(5),$$

where $f^{*}$ is the optimal value of the continuous problem (5).

**Proof.**  Detailed derivations are presented in Appendix A.1.    □

Next, we provide the convergence proof of the RD problem (3) via its discrete problem (7). Let the optimal solution of the continuous problem (3) and the discrete problem (7) be $\left(r^{*},\beta^{*}\right)\;\mathrm{and}\;\left(r^{n},\beta^{n}\right)$ respectively. Since $r^{n}$ has no concrete form, it is not straightforward to prove its convergence. The key idea is a transformation from the convergence of $r^{n}$ to that of a variable $q^{n}$ of certain concrete form, as summarized in Proposition 1 below, which has drawn some inspiration from [25].

**Proposition** **1.** Construct $q^{n}=\sum_{j=1}^{n}A_{j}^{n}\delta_{y_{j}^{n}}$, where $A_{j}^{n}=\int_{I_{j}}dr^{*}$, and $I_{j}$ is defined in Section 2.1. To prove the convergence of the objective value in problem (3), i.e., $F\left(r^{n},\beta^{n}\right)\to F\left(r^{*},\beta^{*}\right)$, we only need to show $F\left(q^{n},{\tilde{\beta }}^{n}\right)\to F\left(r^{*},\beta^{*}\right)$, where ${\tilde{\beta }}^{n}={argmax}_{\beta }F\left(q^{n},\beta \right)$.

**Proof.**  Detailed derivations are presented in Appendix A.2.    □

In the following Lemma 2, we provide the convergence result of ${\tilde{\beta }}^{n}$, as a prelude to the convergence proof for the RD problem.

**Lemma** **2.** *Convergence properties of ${\tilde{\beta }}^{n}$:*

*(1)*  *${\tilde{\beta }}^{n}$ is lower bounded away from zero for any n, i.e., $\exists B_{1}>0$, ${\tilde{\beta }}^{n}\geq B_{1},\forall n$.*
*(2)*  ${\tilde{\beta }}^{n}$ converges to the optimal solution $\beta^{*}$.

**Proof**  (1). By using subsequence analysis [23] and uniform convergence analysis [16], we can obtain the result. A detailed derivation is presented in Appendix A.3.

(2). By using the sequentially compact property [23], the truncation technique and the tools of upper limits and lower limits [16], the convergence can be proven. Detailed derivations are presented in Appendix A.3.    □

At this point, we are ready to establish the convergence of the discrete problem (7) in Theorem 1.

**Proof of Theorem** **1(1).** We now show that $F\left(q^{\;n},{\tilde{\beta }}^{\;n}\right)\to F\left(r^{*},\beta^{*}\right)$. By Lemma 1, $F\left(q^{\;n},\beta^{*}\right)\to F\left(r^{*},\beta^{*}\right)$. Therefore, it suffices to prove that

$$F\left(q^{\;n},{\tilde{\beta }}^{\;n}\right)-F\left(q^{\;n},\beta^{*}\right)⟶0.$$

For this, we notice that

$$\begin{array}{cc} \; & 0\leq F\left(q^{n},{\tilde{\beta }}^{n}\right)-F\left(q^{n},\beta^{*}\right)=-\int \log\frac{\int \exp\left(-{\tilde{\beta }}^{n}\rho \left(x,y\right)\right)dq^{n}\left(y\right)}{\int \exp\left(-\beta^{*}\rho \left(x,y\right)\right)dq^{n}\left(y\right)}dp\left(x\right)-\left({\tilde{\beta }}^{n}-\beta^{*}\right)D. \end{array}$$

We divide the integral into two parts and estimate each of them. For simplicity, denote $H_{r}^{\beta }\left(x\right)=\int \exp\left(-\beta \rho \left(x,y\right)\right)dr\left(y\right)$.

(8) $$\begin{array}{cc} \; & -\int_{∥x∥\leq M}\log\frac{\int \exp\left(-{\tilde{\beta }}^{n}\rho \left(x,y\right)\right)dq^{n}\left(y\right)}{\int \exp\left(-\beta^{*}\rho \left(x,y\right)\right)dq^{n}\left(y\right)}dp\left(x\right)\\ \; & =\int_{∥x∥\leq M}\log\left(\frac{H_{q^{n}}^{\beta^{*}}\left(x\right)-H_{q^{n}}^{{\tilde{\beta }}^{n}}\left(x\right)}{\int \exp\left(-{\tilde{\beta }}^{n}\rho \left(x,y\right)\right)dq^{n}\left(y\right)}+1\right)dp\left(x\right)\\ \; & \leq \int_{∥x∥\leq M}\frac{|H_{q^{n}}^{\beta^{*}}\left(x\right)-H_{q^{n}}^{{\tilde{\beta }}^{n}}\left(x\right)|}{\int \exp\left(-{\tilde{\beta }}^{n}\rho \left(x,y\right)\right)dq^{n}\left(y\right)}dp\left(x\right)\leq \int_{∥x∥\leq M}\frac{|H_{q^{n}}^{\beta^{*}}\left(x\right)-H_{q^{n}}^{{\tilde{\beta }}^{n}}\left(x\right)|}{\delta_{0}}dp\left(x\right), \end{array}$$

where $\delta_{0}$ is a lower bound and

$$\begin{array}{cc} \int \;\;\exp\left(-{\tilde{\beta }}^{n}\rho \left(x,y\right)\right)dq^{n}\left(y\right) & \geq \;\;\int_{∥y∥\leq M}\;\;\exp\left(-B_{0}\rho \left(x,y\right)\right)dq^{n}\left(y\right)\\ \; & \geq \int_{∥y∥\leq M}\exp\left(-B_{0}\rho^{*}\right)dq^{n}\left(y\right)\geq \frac{1}{2}\exp\left(-B_{0}\rho^{*}\right)≜\delta_{0}. \end{array}$$

Here, $B_{0}$ is an upper bound of ${\tilde{\beta }}^{n}$, since it is convergent, *M* is larger than the constant *A* in Lemma 1, and $\rho^{*}$ is a constant equal to $\max_{x,y\in {\left[-M,M\right]}^{d}}\rho \left(x,y\right)$. Using the same derivation as (A7) in the proof of Lemma 2, we have

$$\int \exp\left(-{\tilde{\beta }}^{n}\rho \left(x,y\right)\right)dq^{n}\left(y\right)-\int \exp\left(-\beta^{*}\rho \left(x,y\right)\right)dq^{n}\left(y\right)\;⇉\;0.$$

Thus, when *n* is sufficiently large,

$$\int_{∥x∥\leq M}\frac{|H_{q^{n}}^{\beta^{*}}\left(x\right)-H_{q^{n}}^{{\tilde{\beta }}^{n}}\left(x\right)|}{\delta_{0}}dp\left(x\right)\leq \varepsilon .$$

We obtain

$$\lim_{n}-\int_{∥x∥\leq M}\log\frac{\int \exp\left(-{\tilde{\beta }}^{n}\rho \left(x,y\right)\right)dq^{n}\left(y\right)}{\int \exp\left(-\beta^{*}\rho \left(x,y\right)\right)dq^{n}\left(y\right)}dp\left(x\right)=0.$$

Meanwhile, let $g\left(x\right)=\max_{∥y∥\leq A}\rho \left(x,y\right)$, then

$$\begin{array}{cc} -\int_{∥x∥>M}\log\frac{H_{q^{n}}^{{\tilde{\beta }}^{n}}\left(x\right)}{H_{q^{n}}^{\beta^{*}}\left(x\right)}dp\left(x\right)\; & =\int_{∥x∥>M}\left[-\log\left(H_{q^{n}}^{{\tilde{\beta }}^{n}}\left(x\right)\right)-\log\left(H_{q^{n}}^{\beta^{*}}\left(x\right)\right)\right]dp\left(x\right)\\ \; & \leq \int_{∥x∥>M}\left[-\log\left(\frac{1}{2}e^{-{\tilde{\beta }}^{n}g\left(x\right)}\right)-\log\left(\frac{1}{2}e^{-\beta^{*}g\left(x\right)}\right)\right]dp\left(x\right)\\ \; & =\int_{∥x∥>M}\left[2\log2+{\tilde{\beta }}^{n}g\left(x\right)+\beta^{*}g\left(x\right)\right]dp\left(x\right)\\ \; & \leq \int_{∥x∥>M}\left[2\log2+B_{0}g\left(x\right)+\beta^{*}g\left(x\right)\right]dp\left(x\right). \end{array}$$

Here, we used the fact

$$\begin{array}{cc} \; & H_{q^{n}}^{{\tilde{\beta }}^{n}}\left(x\right)\geq \int_{∥y∥\leq A}\exp\left(-{\tilde{\beta }}^{n}\rho \left(x,y\right)\right)dq^{n}\left(y\right)\\ \; & \geq \int_{∥y∥\leq A}\exp\left(-{\tilde{\beta }}^{n}g\left(x\right)\right)dq^{n}\left(y\right)\geq 1/2\exp\left(-{\tilde{\beta }}^{n}g\left(x\right)\right). \end{array}$$

Combining the two parts and taking the limit n→∞, we obtain

$$\begin{array}{c} \underset{n}{\lim\;\sup}\left[F\left(q^{n},{\tilde{\beta }}^{n}\right)-F\left(q^{n},\beta^{*}\right)\right]\;\\ =\underset{n}{\lim\;\sup}-\int \log\frac{\int \exp\left(-{\tilde{\beta }}^{n}\rho \left(x,y\right)\right)dq^{n}\left(y\right)}{\int \exp\left(-\beta^{*}\rho \left(x,y\right)\right)dq^{n}\left(y\right)}dp\left(x\right)\\ =\underset{n}{\lim\;\sup}\left(\;\;-\;\;\int_{∥y∥>M}\;\;\log\frac{\int \exp\left(-{\tilde{\beta }}^{n}\rho \left(x,y\right)\right)dq^{n}\left(y\right)}{\int \exp\left(-\beta^{*}\rho \left(x,y\right)\right)dq^{n}\left(y\right)}dp\left(x\right)\right)\\ \leq \int_{∥x∥>M}\left[2\log2+B_{0}g\left(x\right)+\beta^{*}g\left(x\right)\right]dp\left(x\right). \end{array}$$

Then by taking the limit of M→∞, we obtain the convergence. Here we used the assumption, i.e., ∫g(x)dp(x)<∞.

To prove the convergence of the solutions, let $\left(\tilde{r},\tilde{\beta }\right)$ be a limit point of the solution sequence ${\left\{\left(r^{n},\beta^{n}\right)\right\}}_{n=1}^{\infty }$. Then there exists a subsequence ${\left\{\left(r^{n_{k}},\beta^{n_{k}}\right)\right\}}_{k=1}^{\infty }$ satisfying $r^{n_{k}}\overset{w}{\to }\tilde{r}$ and $\beta^{n_{k}}\to \tilde{\beta }$. Next, we have

$$F\left(\tilde{r},\tilde{\beta }\right)=\lim_{k}F\left(r^{n_{k}},\beta^{n_{k}}\right)=F\left(r^{*},\beta^{*}\right),$$

since we have proven that $\lim_{n}F\left(r^{n},\beta^{n}\right)=F\left(r^{*},\beta^{*}\right)$. By the optimality property of $\left(r^{*},\beta^{*}\right)$ and $\left(r^{n_{k}},\beta^{n_{k}}\right)$,

$$F\left(\tilde{r},\tilde{\beta }\right)=F\left(r^{*},\beta^{*}\right)\leq F\left(r^{n_{k}},\beta^{*}\right)\leq F\left(r^{n_{k}},\beta^{n_{k}}\right)\leq F\left({\bar{q}}^{n_{k}},\beta^{n_{k}}\right).$$

Here, ${\bar{q}}^{n_{k}}\in W_{n_{k}}$ is a discrete version of $\bar{r}={\mathrm{argmin}}_{r\in W}F\left(r,\tilde{\beta }\right)$, i.e., ${\bar{q}}^{n_{k}}=\sum_{j}\int_{I_{j}}d\bar{r}\;\delta_{y_{j}^{n_{k}}}$. Similarly to the preceding analysis, we have $F\left({\bar{q}}^{n_{k}},\beta^{n_{k}}\right)\to F\left(\bar{r},\tilde{\beta }\right)$. Thus,

$$F\left(\tilde{r},\tilde{\beta }\right)\leq F\left(\bar{r},\tilde{\beta }\right)\leq F\left(r,\tilde{\beta }\right),\;\forall r\in W.$$

This means $\tilde{r}={\mathrm{argmin}}_{r\in W}F\left(r,\tilde{\beta }\right)$ and

$$F\left(\tilde{r},\tilde{\beta }\right)=\min_{r\in W}F\left(r,\tilde{\beta }\right)≜h\left(\tilde{\beta }\right).$$

However, $F\left(\tilde{r},\tilde{\beta }\right)=F\left(r^{*},\beta^{*}\right)=h\left(\beta^{*}\right)$ and $h\left(\beta^{*}\right)=\max_{\beta \geq 0}h\left(\beta \right)$ hold due to the optimality property of $\left(r^{*},\beta^{*}\right)$. So, we obtain that $\tilde{\beta }$ is optimal, i.e., $\tilde{\beta }={\mathrm{argmax}}_{\beta \geq 0}\;h\left(\beta \right)$. Further combining $\tilde{r}={\mathrm{argmin}}_{r\in W}F\left(r,\tilde{\beta }\right)$, we obtain that $\left(\tilde{r},\tilde{\beta }\right)$ is optimal.    □

To prove part (2) in Theorem 1, we first establish the convergence rate for the discrete problems (6).

**Lemma** **3.** *When the probability distribution p of X is supported over ${\left[-M,M\right]}^{d}$, the optimal values $f(r^{n})$ of the discrete problem (6) satisfy the error estimate*

$$|f\left(r^{n}\right)-f^{*}|\;\leq \;Ch,$$

*where C is a constant and $h=2M/n^{\frac{1}{d}}$ is the discretization step size.*

**Proof.**  By refining the estimation in Lemma 1, we obtain the convergence rate. A derivation is shown in Appendix A.4.    □

Correspondingly, the convergence rate of problem (7) can be proved as follows.

**Proof of Theorem** **1(2).** Since r is supported over ${\left[-M,M\right]}^{d}$, we can simply take the equidistant discretization nodes ${\left\{y_{j}^{n}\right\}}_{j=1}^{n}$ from ${\left[-M,M\right]}^{d}$. Due to the inequality (A5) in the proof of Proposition 1,

$$0\leq F\left(r^{n},\beta^{n}\right)-F\left(r^{*},\beta^{*}\right)\leq F\left(q^{n},{\tilde{\beta }}^{n}\right)-F\left(r^{*},\beta^{*}\right).$$

Thus, we only need to evaluate $F\left(q^{n},{\tilde{\beta }}^{n}\right)-F\left(r^{*},\beta^{*}\right)$. Assume for $x,y\in {\left[-M,M\right]}^{d}$ that $e^{-\beta \rho (x,y)}$ and $e^{-\beta \rho (x,y)}\rho \left(x,y\right)$ are Lipschitz continuous with the common Lipschitz constant *L*, for $0<B_{1}\leq \beta \leq B_{0}$.

First, we estimate the convergence rate of ${\tilde{\beta }}^{n}\to \beta^{*}$:

$$\begin{array}{cc} \; & \left|\int \exp\left(-{\tilde{\beta }}^{n}\rho \left(x,y\right)\right)dq^{n}\left(y\right)-\int \exp\left(-{\tilde{\beta }}^{n}\rho \left(x,y\right)\right)dr^{*}\left(y\right)\right|\\ \; & \leq \sum_{i}\int_{I_{i}}\left|\exp\left(-{\tilde{\beta }}^{n}\rho \left(x,y\right)\right)-\exp\left(-{\tilde{\beta }}^{n}\rho \left(x,y_{i}^{n}\right)\right)\right|dr^{*}\left(y\right)\\ \; & \leq \sum_{i}\int_{I_{i}}L∥y-y_{i}^{n}∥dr^{*}\left(y\right)\leq Lh/2. \end{array}$$

Here, we used the fact ${⋃}_{i=1}^{n}I_{i}⊇{\left[-M,M\right]}^{d}$, and the bound on ${\tilde{\beta }}^{n}$, i.e., $0<B_{1}\leq {\tilde{\beta }}^{n}\leq B_{0}$.

Similarly,

$$|\int \exp\left(-{\tilde{\beta }}^{n}\rho \left(x,y\right)\right)\rho \left(x,y\right)dq^{n}\left(y\right)-\int \exp\left(-{\tilde{\beta }}^{n}\rho \left(x,y\right)\right)\rho \left(x,y\right)dr^{*}\left(y\right)|\leq Lh/2.$$

Next, as in the proof of Theorem 1(1), we obtain the lower bounds

$$\int e^{-{\tilde{\beta }}^{\;n}\rho \left(x,y\right)}\;dq^{\;n}\left(y\right)\;\geq \;\delta_{0},\;\int e^{-{\tilde{\beta }}^{\;n}\rho \left(x,y\right)}\;dr^{*}\left(y\right)\;\geq \;\delta_{0}.$$

Moreover, since ${\tilde{\beta }}^{\;n}\geq B_{1}>0$ and $e^{-\beta \rho }$ is decreasing in β,

$$\begin{array}{cc} \int e^{-{\tilde{\beta }}^{\;n}\rho \left(x,y\right)}\;\rho \left(x,y\right)\;dr^{*}\left(y\right)\; & \leq \int e^{-B_{1}\rho \left(x,y\right)}\;\rho \left(x,y\right)\;dr^{*}\left(y\right)\leq \int M_{1}\;dr^{*}\left(y\right)\;=\;M_{1}, \end{array}$$

where $M_{1}$ is the upper bound on the function $\exp(-B_{1}t)t,t\geq 0$.

Now, since $G_{r^{*}}\left(\beta^{*}\right)=0=G_{q^{n}}\left({\tilde{\beta }}^{n}\right)$, we have the following estimate:

$$\begin{array}{cc} |G_{r^{*}}\left({\tilde{\beta }}^{n}\right)-G_{r^{*}}\left(\beta^{*}\right)| & =|G_{r^{*}}\left({\tilde{\beta }}^{n}\right)-G_{q^{n}}\left({\tilde{\beta }}^{n})\right|\\ \; & \leq \int_{∥x∥\leq M}|\frac{\left(\int \exp\left(-{\tilde{\beta }}^{n}\rho \left(x,y\right)\right)\rho \left(x,y\right)dq^{n}\left(y\right)\right)}{\left(\int \exp\left(-{\tilde{\beta }}^{n}\rho \left(x,y\right)\right)dq^{n}\left(y\right)\right)}\\ \; & \;-\frac{\left(\int \exp\left(-{\tilde{\beta }}^{n}\rho \left(x,y\right)\right)\rho \left(x,y\right)dr^{*}\left(y\right)\right)}{\left(\int \exp\left(-{\tilde{\beta }}^{n}\rho \left(x,y\right)\right)dr^{*}\left(y\right)\right)}|dp\left(x\right)\\ \; & =\int_{∥x∥\leq M}|\frac{b_{1}}{a_{1}}-\frac{b_{2}}{a_{2}}|dp\left(x\right)=\int_{∥x∥\leq M}\frac{|a_{2}b_{1}-a_{1}b_{2}|}{a_{1}a_{2}}dp\left(x\right)\\ \; & \leq \int_{∥x∥\leq M}\frac{a_{2}|b_{1}-b_{2}|+b_{2}\left|a_{2}-a_{1}\right|}{a_{1}a_{2}}dp\left(x\right)\\ \; & \leq \int_{∥x∥\leq M}\left(\frac{Lh}{2\delta_{0}}+\frac{M_{1}Lh/2}{\delta_{0}^{2}}\right)dp\left(x\right)\\ \; & \leq \frac{Lh}{2\delta_{0}}+\frac{M_{1}Lh/2}{\delta_{0}^{2}}=O\left(h\right), \end{array}$$

wherein for the simplicity of notation, we write $a_{1},a_{2},b_{1},b_{2}$ for the respective integrals.

Next, we provide the estimate of ${\tilde{\beta }}^{n}-\beta^{*}$. Let $-L_{1}=G_{r^{*}}^{\prime }\left(\beta^{*}\right)<0$. Then there exists $\delta_{2}>0$ such that, for all $\beta \in (\beta^{*}-\delta_{2},\beta^{*}+\delta_{2})$,

$$G_{r^{*}}^{\prime }\left(\beta \right)\leq -\frac{L_{1}}{2},$$

and ${\tilde{\beta }}^{n}$ lies in this neighborhood once *n* is sufficiently large. Therefore, by the Lagrange mean value theorem,

$$|G_{r^{*}}\left({\tilde{\beta }}^{n}\right)-G_{r^{*}}\left(\beta^{*}\right)|=|G_{r^{*}}^{\prime }\left(\zeta \right)|\;|{\tilde{\beta }}^{n}-\beta^{*}|\geq \frac{L_{1}}{2}\left|{\tilde{\beta }}^{n}-\beta^{*}\right|.$$

Here, ζ is a real number between ${\tilde{\beta }}^{n}$ and $\beta^{*}$. Thus,

$$|{\tilde{\beta }}^{n}-\beta^{*}|\leq \frac{2}{L_{1}}\left|G_{r^{*}}\left({\tilde{\beta }}^{n}\right)-G_{r^{*}}\left(\beta^{*}\right)\right|=O\left(h\right).$$

Now we provide the convergence rate of $F\left(q^{n},{\tilde{\beta }}^{n}\right)-F\left(r^{*},\beta^{*}\right)$:

$$|F\left(q^{n},{\tilde{\beta }}^{n}\right)-F\left(r^{*},\beta^{*}\right)|\leq |F\left(q^{n},{\tilde{\beta }}^{n}\right)-F\left(q^{n},\beta^{*}\right)|+|F\left(q^{n},\beta^{*}\right)-F\left(r^{*},\beta^{*}\right)|.$$

By conducting the following evaluation

$$\begin{array}{cc} \; & \left|\int \exp\left(-\beta^{*}\rho \left(x,y\right)\right)dq^{n}\left(y\right)-\int \exp\left(-{\tilde{\beta }}^{n}\rho \left(x,y\right)\right)dq^{n}\left(y\right)\right|\\ \; & \leq \int e^{-\beta^{*}\rho \left(x,y\right)}\left|e^{\left(\beta^{*}-{\tilde{\beta }}^{n}\right)\rho \left(x,y\right)}-1\right|dq^{n}\left(y\right)\\ \; & \leq \int_{∥y∥\leq M}\left(e^{|\beta^{*}-{\tilde{\beta }}^{n}|\rho \left(x,y\right)}-1\right)\;dq^{n}\left(y\right)\\ \; & \leq \int_{∥y∥\leq M}\left(e^{|\beta^{*}-{\tilde{\beta }}^{n}|\rho^{*}}-1\right)\;dq^{n}\left(y\right)\leq \left(e^{|\beta^{*}-{\tilde{\beta }}^{n}|\rho^{*}}-1\right)\\ \; & \leq 2|\beta^{*}-{\tilde{\beta }}^{n}|\rho^{*}=O\left(h\right),\;\mathrm{when}\;n\;\mathrm{is}\;\mathrm{sufficiently}\;\mathrm{large}, \end{array}$$

where $\rho^{*}=\max_{x,y\in {\left[-M,M\right]}^{d}}\rho \left(x,y\right)$, we thus have the following estimate using the derivation in (8):

$$\begin{array}{cc} \; & 0\leq F\left(q^{n},{\tilde{\beta }}^{n}\right)-F\left(q^{n},\beta^{*}\right)\\ \; & \leq \int_{∥x∥\leq M}\frac{\left|H_{q^{n}}^{\beta^{*}}\left(x\right)-H_{q^{n}}^{{\tilde{\beta }}^{n}}\left(x\right)\right|}{\delta_{0}}dp\left(x\right)-\left({\tilde{\beta }}^{n}-\beta^{*}\right)D\\ \; & \leq \int_{∥x∥\leq M}O\left(h\right)/\delta_{0}\;dp\left(x\right)+O\left(h\right)=O\left(h\right), \end{array}$$

where we denote $H_{r}^{\beta }\left(x\right)=\int \exp\left(-\beta \rho \left(x,y\right)\right)dr\left(y\right)$. Meanwhile, due to Lemma 3, $|F\left(q^{n},\beta^{*}\right)-F\left(r^{*},\beta^{*})|=O(h\right)$. Thus, we obtain the convergence rate $\left|F(r^{n},\beta^{n}\right)-F(r^{*},\beta^{*})|=O\left(h\right)=O\left(1/n^{\frac{1}{d}}\right).$    □

**Remark** **1.** *We first want to clarify that Theorem 1, as presented here, is specifically centered on the CBA algorithm. Additionally, it should be noted that corresponding results for the BA algorithm are also covered: both Lemma 1 and Lemma 3 entail the same conclusions for the BA algorithm.*

### 2.3. Numerical Verfication

**Figure 1:**
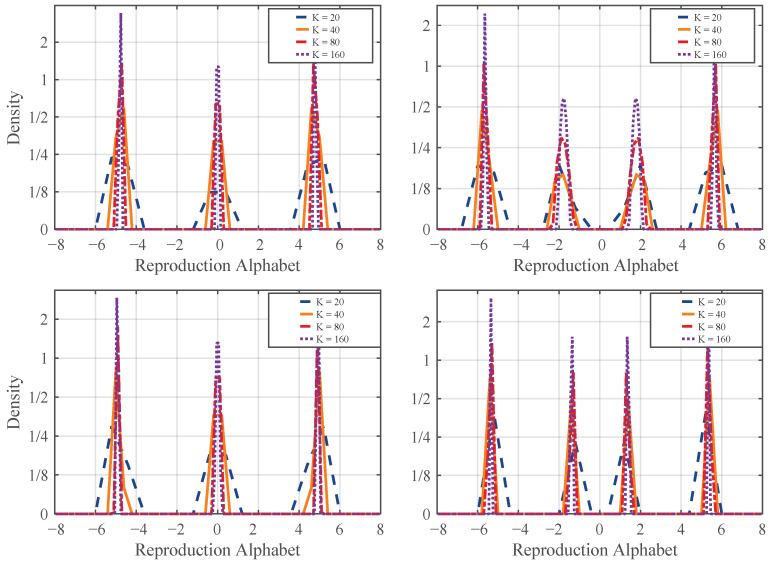
The discrete optimal reproduction distribution produced by the BA algorithm for slope β=0.1 (**upper left**) and β=0.2 (**upper right**), and by the CBA algorithm for target distortion D=4 (**lower left**) and D=3 (**lower right**). For clearer visualization, the $\log_{2}$ scale of the density is used on the vertical axis.

We conduct experiments on a uniform source to confirm the convergence shown in this section. We consider the uniform source on interval [−8,8] and solve the corresponding discrete problems with different discretization parameters, with the node number varying according to n=20,40,80,160 for the discretization of *Y*. In order to ensure accuracy for evaluating the integrals with respect to p(x), we fix a sufficiently large node number m=300 for *X*. We use both the BA and CBA algorithms to solve the discrete problems (6) and (7), respectively. The corresponding results of the reproduction distribution are illustrated in Figure 1.

It has been well known (see, i.e., [17]) that the optimal reproduction distribution of the uniform source is discrete. From Figure 1, the convergence of the solutions of discrete problems to discrete reproduction distributions is clearly demonstrated, as the discretization becomes finer progressively.

## 3. Discrete RD Algorithms and Their ε-Accuracy Analysis

In the previous section, we have shown that for computing the RD function of a continuous source, as discretization step size asymptotically vanishes, solutions to discrete problems converge to those of the continuous problem. Discrete problems are typically solved via BA [10,11] and CBA [12] algorithms. In this section, we review these algorithms, and provide estimates on their required amount of arithmetic operations for attaining ε-accuracy within the solution of the continuous problem.

### 3.1. BA Algorithm

As a classical numerical method for computing the RD function, the BA algorithm [10,11] minimizes the RD Lagrangian with a fixed penalty parameter. Concretely, for a fixed penalty β>0, it minimizes

$$L_{\mathrm{RD}}^{\beta }\left(P_{Y|X}\right)≜I\left(X;Y\right)+\beta \;E_{P_{XY}}\left[d(X,Y)\right].$$

Geometrically, β is the slope of a tangent line to the RD curve R(D); hence, minimizing $L_{\mathrm{RD}}^{\beta }$ at a fixed β yields a conditional distribution $P_{Y|X}^{*}$ associated with the point of tangency $\left(D_{\lambda },R_{\lambda }\right)$ on the RD curve [26].

Write w(y|x) for the conditional probability $P_{Y|X}$, and r(y) for the marginal distribution of *Y*. The BA algorithm alternates between the two variables w(y|x) and r(y) in the following way [10]:

$$\begin{array}{cc} w(y|x) & =\left(r\left(y\right)e^{-\beta \rho (x,y)}\right)/\int e^{-\beta \rho (x,y)}r\left(y\right)dy,\\ r(y) & =\int p(x)w(y|x)dx. \end{array}$$

After discretization, the alternating iterations can be reformulated as:

(9a) $$\begin{array}{cc} w_{ij} & =\left(r_{j}e^{-\beta \rho_{ij}}\right)/\sum_{j=1}^{n}e^{-\beta \rho_{ij}}r_{j},\;i=1,2\cdots m,\;j=1,2\cdots n, \end{array}$$

(9b) $$\begin{array}{cc} r_{j} & =\sum_{i=1}^{m}p_{i}w_{ij},\;j=1,2\cdots n, \end{array}$$

where $w_{ij}=w\left(y_{j}|x_{i}\right)h,\;p_{i}=p\left(x_{i}\right)h,\;r_{j}=r\left(y_{j}\right)h,\rho_{ij}=\rho \left(x_{i},y_{j}\right)$, and *h* is the discretization step size of uniform discretization.

It has been shown in the literature (see, e.g., [26] (Chapter 8)) that, for discrete sources, the iterative procedure of the BA algorithm converges to the RD function at rate O(1/n). Furthermore, as shown in [13] (Theorem 2, Equation (96)), the BA algorithm needs O(logn/ε) iterations to achieve ε-accuracy. In each iteration, the BA algorithm performs two matrix–vector multiplications, involving O(mn) arithmetic operations. Moreover, the space cost amounts to O(mn) since we need to store $w_{ij}$ and $r_{j},\;i,=1,2,\cdots ,m,\;j=1,2\cdots n$.

Based on the convergence rate analysis in Lemma 3, we conduct a complexity analysis to estimate the computational cost of solving the continuous problem (5) to achieve ε-accuracy via the BA algorithm.

**Theorem** **2.** *To ensure ε-accuracy for computing the continuous RD function, the BA algorithm needs $O\left(\frac{m|\log\varepsilon |}{\varepsilon^{d+1}}\right)$ arithmetic operations. Here, m is the number of discretization nodes of X when conducting numerical integration and d is the dimension of the reproduction space Y.*

**Proof.**  By Theorem 3, to ensure that $f\left(r^{n}\right)-f\left(r^{*}\right)\leq \varepsilon$, the sample size *n* must satisfy $1/n^{1/d}∼\varepsilon$, i.e., $n∼\varepsilon^{-d}$. Subsequently, the BA algorithm is applied to solve the associated discrete problem up to an error tolerance at level ε. As shown in [13] (Theorem 2, Equation (96)), the BA algorithm needs O(logn/ε) iterations to achieve ε-accuracy. In each iteration, the BA algorithm alternates between $w(y_{i}^{n}|x)$ and $r(y_{i}^{n})$ in the following way:

$$\begin{array}{cc} w(y_{i}^{n}|x) & =\left(r\left(y_{i}^{n}\right)e^{-\beta \rho (x,y_{i}^{n})}\right)/\sum_{i=1}^{n}e^{-\beta \rho (x,y_{i}^{n})}r\left(y_{i}^{n}\right),\\ r(y_{i}^{n}) & =\int p\left(x\right)w\left(y_{i}^{n}|x\right)dx. \end{array}$$

Let $x_{1},x_{2}\cdots x_{m}$ be the nodes of numerical integration with respect to *x*. When computing $w(y_{i}^{n}|x)$, we need to perform matrix–vector multiplication

$$\sum_{i=1}^{n}e^{-\beta \rho (x_{j},y_{i}^{n})}r\left(y_{i}^{n}\right),\;j=1,2\cdots m,$$

and it involves O(mn) arithmetic operations. The total computation cost for $w(y_{i}^{n}|x)$ is O(mn). When computing $r(y_{i}^{n})$, we need to perform numerical integration,

$$\int p\left(x\right)w\left(y_{i}^{n}|x\right)dx∼\sum_{j=1}^{m}A_{j}p\left(x_{j}\right)w\left(y_{i}^{n}|x_{j}\right),\;i=1,2\cdots n,$$

where $A_{j}$ are the numerical integration coefficients. This is a matrix–vector multiplication, and it involves O(mn) arithmetic operations. Thus, the computational cost for each iteration in the BA algorithm is O(mn). Hence, the total computational cost is $O\left(mn\log n/\varepsilon \right)=O(m|\log\varepsilon |/\varepsilon^{d+1})$.    □

### 3.2. CBA Algorithm

Inspired by the connection between OT and RD [27,28], Chen et al. [12] introduced the CBA algorithm [12], which explicitly incorporates the expected distortion constraint of the RD function. This allows the Lagrange multiplier β to be updated via a one-dimensional root-finding step to meet a target distortion *D* in each iteration, thereby solving the original RD problem (1) directly, without sweeping the entire RD curve as in the BA algorithm. Specifically, when alternating between the two variables w(y|x) and r(y), the CBA algorithm updates the multiplier β (see [12] for further details):

$$\begin{array}{cc} \mathrm{Solve}\;G(\beta ) & ≜\int \frac{\left(\int e^{-\beta \rho (x,y)}\rho \left(x,y\right)r\left(y\right)dy\right)}{\left(\int e^{-\beta \rho (x,y)}r\left(y\right)dy\right)}p\left(x\right)dx-D=0,\\ w(y|x) & =\left(r\left(y\right)e^{-\beta \rho (x,y)}\right)/\int e^{-\beta \rho (x,y)}r\left(y\right)dy,\\ r(y) & =\int p(x)w(y|x)dx, \end{array}$$

where β can be efficiently evaluated by Newton’s method since G(β) is monotone, as shown in [12]. After discretization, the iterative procedure turns into:

(10a) $$\begin{array}{cc} \mathrm{Solve}\;G(\beta ) & ≜\sum_{i=1}^{m}\frac{\left(\sum_{j=1}^{n}e^{-\beta \rho_{ij}}\rho_{ij}r_{j}\right)}{\left(\sum_{j=1}^{n}e^{-\beta \rho_{ij}}r_{j}\right)}p_{i}-D=0, \end{array}$$

(10b) $$\begin{array}{cc} w_{ij} & =\left(r_{j}e^{-\beta \rho_{ij}}\right)/\sum_{j=1}^{m}e^{-\beta \rho_{ij}}r_{j},\;i=1,2,\cdots ,m,\;j=1,2\cdots n, \end{array}$$

(10c) $$\begin{array}{cc} r_{j} & =\sum_{i=1}^{m}p_{i}w_{ij},\;j=1,2\cdots n, \end{array}$$

where $w_{ij}=w\left(y_{j}|x_{i}\right)h,\;p_{i}=p\left(x_{i}\right)h,\;r_{j}=r\left(y_{j}\right)h,\rho_{ij}=\rho \left(x_{i},y_{j}\right)$ and *h* is the discretization step sizes. As shown in [12], for discrete sources, the iterative procedure of the CBA algorithm also converges to the RD function at rate O(1/n). Note that differently from the BA algorithm which minimizes the RD Lagrangian for a given multiplier λ, the CBA algorithm directly reaches the RD function for a given target distortion *D*. By the analysis in [12], in each iteration, the CBA algorithm takes $O\left(mn(1+\log|\log\varepsilon |)\right)$ arithmetic operations to achieve ε-accuracy for solving β, while the space cost is also O(mn).

Analogous to Theorem 2, we provide the complexity analysis to estimate the computational cost of solving the continuous problem (3) to achieve ε-accuracy via the CBA algorithm.

**Theorem** **3.** *To ensure ε-accuracy for computing the continuous RD function, the CBA algorithm needs $O\left(\frac{m|\log\varepsilon |}{\varepsilon^{d+1}}\left(1+\log|\log\varepsilon |\right)\right)$ arithmetic operations. Here, m is the number of discretization nodes of X when conducting numerical integration and d is the dimension of the reproduction space Y.*

**Proof.**  By Lemma 3, to ensure that $F\left(r^{n},\beta^{n}\right)-F\left(r^{*},\beta^{*}\right)\leq \varepsilon$, the sample size *n* must satisfy $1/n^{1/d}∼\varepsilon$, i.e., $n∼1/\varepsilon^{d}$. Then when using the CBA algorithm to solve the discrete problem within ε error tolerance, as shown in [12], we need $O\left(\frac{mn\log n}{\varepsilon }\left(1+\log|\log\varepsilon |\right)\right)$ arithmetic operations to achieve ε-accuracy. Thus, the computational cost is

$$O\left(\frac{mn\log n}{\varepsilon }\left(1+\log|\log\varepsilon |\right)\;\right)=O\left(\frac{m|\log\varepsilon |}{\varepsilon^{d+1}}\left(1+\log|\log\varepsilon |\right)\right),$$

thereby completing the proof.    □

## 4. Acceleration Techniques for the CBA Algorithm

The previous section detailed the computational cost for discrete algorithms (i.e., BA and CBA) to obtain ε-accurate RD solutions for continuous sources. To this end, we exploit the structure of specific distortion measures (i.e., squared-error and absolute-error) for developing fast algorithms. For clarity, we present the algorithms for the case of m=n; the case of m≠n follows with minor modifications.

### 4.1. Squared-Error Distortion

We begin with the one-dimensional case, i.e., d=1. Following the discretization form introduced in Section 3, we have for the squared-error distortion $\rho \left(x,y\right)={\left(x-y\right)}^{2}$:

$$\rho_{ij}={\left(x_{i}-y_{j}\right)}^{2}=h^{2}{\left(i-j\right)}^{2},\;K_{ij}≜e^{-\beta \rho_{ij}}=e^{-\beta h^{2}{\left(i-j\right)}^{2}}.$$

For simplicity, we denote by $D_{\rho }$ the matrix whose (i,j)-entry is $\rho_{ij}$. Noting that $K_{ij}$ only depends on the difference between its indices, consequently, the two weighted sums in the CBA iterative procedure become linear convolutions and can be implemented in O(mlogm) time by FFT with zero padding.

To begin, we clarify the notation and definitions. We refer to ∗ as linear convolution, and “./” and “⊙” denote element-wise division and multiplication, respectively. Here we denote the standard FFT operation and the standard inverse FFT operation as FFT and IFFT, respectively.

For a given matrix *K*, $K_{d}$ denotes the even extension of its first column, constructed by concatenating the first column of *K* with the reversed first row of *K*, excluding the first entry. Thus, we can compute each convolution K∗v in O(mlogm) time via the FFT [29,30]. Specifically, the vector *v* is zero-padded [31] to length L=2m−1 and $K_{d}$ is constructed; their discrete Fourier transforms are then computed, multiplied component-wise in the Fourier domain, and followed by the inverse transform. The leading *m* coefficients are retained to recover the linear convolution. This process is referred to as the function FFT_conv(K,v).

Substituting (10b) into (10c), the iterations of the CBA algorithm turn into:

(11a) $$\begin{array}{cc} \mathrm{Solve}\;G(\beta ) & =\sum_{i=1}^{m}p_{i}\;\frac{\sum_{j}K_{ij}\;\rho_{ij}\;r_{j}}{\sum_{j}K_{ij}\;r_{j}}-D=0, \end{array}$$

(11b) $$\begin{array}{cc} r_{j} & =r_{j}\sum_{i=1}^{m}\frac{p_{i}K_{ij}}{\sum_{j=1}^{m}K_{ij}r_{j}},\;j=1,2\cdots m. \end{array}$$

We adopt the notation $K^{\left(0\right)}=K,K^{\left(2\right)}=K⊙D_{\rho },K^{\left(4\right)}=K⊙D_{\rho }⊙D_{\rho }$, and let $a=K^{\left(0\right)}*r$ and $u=K^{\left(2\right)}*r$. Then, β can be updated by

(12) $$\mathrm{Solve}\;G\left(\beta \right)=\sum_{i=1}^{m}p_{i}\;\frac{u_{i}}{a_{i}}-D=0.$$

Differentiating G(β) yields

$$G^{\prime }\left(\beta \right)=\sum_{i=1}^{m}p_{i}\;\frac{u_{i}^{2}-a_{i}v_{i}}{a_{i}^{2}},\;v=K^{\left(4\right)}*r.$$

Note that $G^{\prime }\left(\beta \right)$ and G(β) require three FFT-based convolutions (a,u,v), which can be calculated within O(mlogm) arithmetic operations. Thus, each Newton’s iteration involves O(mlogm) arithmetic operations. Due to the locally quadratic convergence of Newton’s method, we need log|logε| iterations to achieve ε-accuracy. Hence, we only need O(mlog|logε|) arithmetic operations to implement (11a). Additionally, the update of (11b) requires two FFT-based convolutions,

(13) $$a=K^{\left(0\right)}*r,\;b=p./a,\;s=K^{\left(0\right)}*b,\;r\leftarrow r⊙s,$$

which can also be calculated within O(mlogm) arithmetic operations. Therefore, in each iteration of (11), it involves $O\left(m\log m(1+\log|\log\varepsilon |)\right)$ arithmetic operations. Moreover, due to [12] (Theorem 3.2), we need O(logm/ε) iterations. Therefore, the total computational cost of our method is $O\left(\frac{m{\left(\log m\right)}^{2}}{\varepsilon }\left(1+\log|\log\varepsilon |\right)\right)$. Since m∼1/ε is needed to ensure the accuracy of numerical integration, the computational cost turns out to be $O\left(\frac{{\left|\log\varepsilon \right|}^{2}}{\varepsilon^{2}}\left(1+\log|\log\varepsilon |\right)\right)$ to achieve ε-accuracy. Meanwhile, the space cost is O(m), since only O(m) variables are stored. The corresponding pseudo-code is shown in Algorithm 1:
**Algorithm 1** FFT-CBA for squared-error distortion (1D)**Input:** 
Source distribution $p_{i}$, target distortion *D*, maximum number of iterations max_iter**Output:** 
Minimal value $\sum_{i=1}^{m}\sum_{j=1}^{m}p_{i}w_{ij}\log\frac{w_{ij}}{r_{j}}$  1:**Initialization:** $r=1_{m}/m$, β=0  2:**for** t=1:max_iter **do**  3:       **while** |G(β)|≥ε **do**  4:            Calculate $K^{\left(0\right)},K^{\left(2\right)},K^{\left(4\right)}$ and $K_{d}^{\left(0\right)},K_{d}^{\left(2\right)},K_{d}^{\left(4\right)}$  5:            Calculate ${\hat{K}}^{\left(q\right)}=\mathrm{FFT}\left(K^{\left(q\right)}\right)$, q=0,2,4  6:            $a=FFT\_conv(K^{\left(0\right)},r)$  7:            $u=FFT\_conv(K^{\left(2\right)},r)$  8:            $v=FFT\_conv(K^{\left(4\right)},r)$  9:            $G\leftarrow \sum_{i}p_{i}\;u_{i}/a_{i}-D$,    $G^{\prime }\leftarrow \sum_{i}p_{i}\;\left(u_{i}^{2}-a_{i}v_{i}\right)/a_{i}^{2}$10:            $\beta \leftarrow \beta -G/G^{\prime }$11:       $a=FFT\_conv(K^{\left(0\right)},r)$12:       b←p./a13:       $s=FFT\_conv(K^{\left(0\right)},b)$14:       r←r⊙s

After obtaining the optimal variables β and r via iterations (11), we can calculate the rate *R* for a given target distortion *D* directly:

$$R=-\beta D-\sum_{i=1}^{m}p_{i}\log\;\left(\sum_{j=1}^{m}K_{ij}r_{j}\right).$$

Here, we can apply an additional FFT to compute *R*, allowing us to obtain *R* using only O(mlogm) arithmetic operations.

**Remark** **2.** *In the implementation, the matrices $K^{\left(0\right)},K^{\left(2\right)},K^{\left(4\right)}$ do not need to be computed explicitly. Instead, we first convert the distance matrix $D_{\rho }$ into vector form and then treat this vector as a constant that is combined with the iteration-dependent β via vector-wise operations in each iteration.*

Next, we consider the case of multiple dimensions. Let $X=Y=R^{d}$ with $\rho \left(x,y\right)={∥x-y∥}_{2}^{2}$. Under a uniform grid of side *M* per dimension, $m=M^{d}$, we have

$$\rho_{ij}=h^{2}\sum_{k=1}^{d}{\left(i_{k}-j_{k}\right)}^{2},\;K_{ij}=e^{-\beta h^{2}\sum_{k=1}^{d}{\left(i_{k}-j_{k}\right)}^{2}}=\prod_{k=1}^{d}e^{-\beta h^{2}{\left(i_{k}-j_{k}\right)}^{2}}.$$

Note that each convolution K∗v can be decomposed into *d* successive one-dimensional FFT-based convolutions:

$$K*v=K^{\left(1\right)}{*}_{1}\left(K^{\left(2\right)}{*}_{2}\left(\cdots \left(K^{\left(d\right)}{*}_{d}v\right)\cdots \right)\right),$$

where ${*}_{k}$ denotes the one-dimensional convolution along the *k*-th coordinate axis, and $K_{i_{k}j_{k}}^{\left(k\right)}=\exp\left(-\beta h^{2}{\left(i_{k}-j_{k}\right)}^{2}\right).$ Therefore, the arithmetic operations per iteration are $O\left(m\log M\right)$. Similarly to the analysis of the one-dimensional case, the complexity of the accelerated CBA algorithm under squared-error distortion with dimension *d* is

$$O\;\left(\frac{m\log m\log m}{\varepsilon }\;\left(1+\log|\log\varepsilon |\right)\right)=O\;\left(\frac{{\left|\log\varepsilon \right|}^{2}}{\varepsilon^{d+1}}\;\left(1+\log|\log\varepsilon |\right)\right),$$

where $m=M^{d}∼\varepsilon^{-d}$.

**Example** **1.** *We evaluate our accelerated algorithms on one-dimensional continuous sources and compare them with the baseline of the CBA algorithm without acceleration. We consider two classical examples—Gaussian and Laplacian sources—and evaluate each source under squared-error distortion measures. We set the accuracy to ε=0.001, yielding m=n=1000 discretization nodes for X and Y, and we cap the number of iterations at max_iter=1000. The discretization nodes are uniformly sampled from the interval [−10,10].*

*Under the squared-error distortion setting, the computational time and the discrepancy in the output rate R between the two algorithms for various values of β are reported in Table 1. As shown in the table, We can see that the Fast CBA algorithm has an overwhelming advantage in computational speed. Moreover, the rates R obtained by the two algorithms are almost identical.*

**Figure 2:**
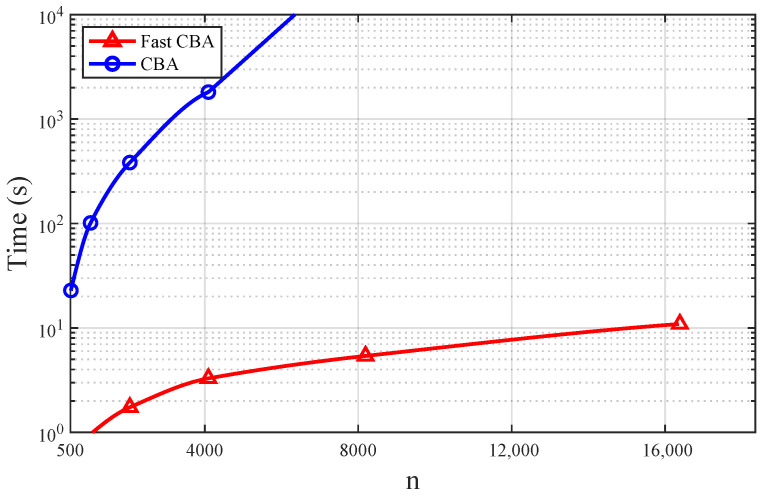
The comparison of computational time between the Fast CBA algorithm and the CBA algorithm with different numbers of grid points *n* under square-error distortion.

*Fixing D=0.4, by data fitting, we can see that the empirical complexity of the Fast CBA algorithm is $O(n^{0.86})$, while that of the CBA algorithm is $O(n^{2.08})$; see Figure 2 for illustration.*

### 4.2. Absolute-Error Distortion

#### 4.2.1. One-Dimension Case

First, we consider the d=1 case X=Y=R with distortion measure ρ(x,y)=|x−y|. Under a uniform discretization with step size *h*, this yields $\rho_{ij}=|x_{i}-y_{j}|=|i-j|h$. For convenience, we denote $\lambda =e^{-\beta h}$.

Substituting (10b) into (10c), the iterations of the CBA algorithm become:

(14a) $$\begin{array}{cc} \mathrm{Solve}\;G(\beta ) & ≜\sum_{i=1}^{m}\frac{\left(\sum_{j=1}^{m}\lambda^{\left|i-j\right|}\left|i-j\right|hr_{j}\right)}{\left(\sum_{j=1}^{m}\lambda^{\left|i-j\right|}r_{j}\right)}p_{i}-D=0, \end{array}$$

(14b) $$\begin{array}{cc} r_{j} & =r_{j}\sum_{i=1}^{m}\frac{p_{i}\lambda^{\left|i-j\right|}}{\sum_{j=1}^{m}\lambda^{\left|i-j\right|}r_{j}},\;j=1,2\cdots m. \end{array}$$

Although the equation G(β)=0 cannot be solved exactly, the prescribed ε-accuracy of the final result is guaranteed, as established in [12] (Theorem 3.2). In practice, Newton’s method for solving (14a) is implemented via the following iterative scheme until the stopping criterion |G(β)|≤ε is satisfied.

(15) $$\beta \leftarrow \beta -G\left(\beta \right)/G^{\prime }\left(\beta \right).$$

By calculation, $G^{\prime }\left(\beta \right)$ can be written as the following form:

$$G^{\prime }\left(\beta \right)=\sum_{i=1}^{m}p_{i}\frac{-\left(\sum_{j=1}^{m}\lambda^{\left|i-j\right|}{\left|i-j\right|}^{2}h^{2}r_{j}\right)\left(\sum_{j=1}^{m}\lambda^{\left|i-j\right|}r_{j}\right)+{\left(\sum_{j=1}^{m}\lambda^{\left|i-j\right|}\left|i-j\right|hr_{j}\right)}^{2}}{{\left(\sum_{j=1}^{m}\lambda^{\left|i-j\right|}r_{j}\right)}^{2}}.$$

Therefore, the main arithmetic complexity in the update of β lies in the computation of $\sum_{j=1}^{m}\lambda^{\left|i-j\right|}\left|i-j\right|r_{j}$ and $\sum_{j=1}^{m}\lambda^{\left|i-j\right|}{\left|i-j\right|}^{2}r_{j}$.

Since λ is related to β, we denote $a_{i}=\sum_{j=1}^{m}\lambda^{\left|i-j\right|}r_{j}$. Let

$$b_{i}=\sum_{j=1}^{i}\lambda^{\left|i-j\right|}r_{j},\;c_{i}=\sum_{j=i+1}^{m}\lambda^{\left|i-j\right|}r_{j},\;a_{i}=b_{i}+c_{i}.$$

Similarly to the treatment in [20], we have the following recursive formulae:

$$\begin{array}{cc} \; & b_{i+1}=\lambda b_{i}+r_{i+1},\;i=1,2\cdots m-1,\\ \; & c_{i-1}=\lambda \left(c_{i}+r_{i}\right),\;i=m,m-1\cdots 2, \end{array}$$

with initial values $b_{1}=r_{1},\;c_{m}=0$.

By exploiting the above recursion, the sequences ${\left\{b_{i}\right\}}_{i=1}^{m}$ and ${\left\{c_{i}\right\}}_{i=1}^{m}$ can be computed sequentially. Each step requires only one multiplication and one addition. Consequently, the total number of arithmetic operations needed to compute $b_{i}$ and $c_{i}$, for i=1,2,⋯,m, is O(m). Therefore, the evaluation of $a_{i}$ can be completed in O(m) arithmetic operations.

Next, we leverage dynamic programming [32] to compute the sums $\sum_{j=1}^{m}\lambda^{\left|i-j\right|}\left|i-j\right|r_{j}$ and $\sum_{j=1}^{m}\lambda^{\left|i-j\right|}{\left|i-j\right|}^{2}r_{j}$ efficiently. Note that these sums differ from $\sum_{j=1}^{m}\lambda^{\left|i-j\right|}r_{j}$, so we cannot directly adopt the approach in [20]. However, with appropriate adjustments, we can also compute them in linear complexity. We denote

$$u_{i}=\sum_{j=1}^{i}\lambda^{\left|i-j\right|}\left|i-j\right|r_{j},\;v_{i}=\sum_{j=i+1}^{m}\lambda^{\left|i-j\right|}\left|i-j\right|r_{j}.$$

Then,

$$\begin{array}{c} \lambda u_{i}=u_{i+1}-\lambda b_{i},\;\lambda v_{i}=v_{i-1}-\lambda r_{i}-\lambda c_{i}. \end{array}$$

Thus, we obtain the following recursive formulae:

$$\begin{array}{cc} \; & u_{i+1}=\lambda \left(u_{i}+b_{i}\right),\;i=1,2\cdots m-1,\\ \; & v_{i-1}=\lambda \left(v_{i}+c_{i}+r_{i}\right),\;i=m,m-1\cdots 2, \end{array}$$

with initial values $u_{1}=0,\;v_{m}=0$. In each recursion, we perform three additions and two multiplications. Since ${\left\{b_{i}\right\}}_{i=1}^{m}$ and ${\left\{c_{i}\right\}}_{i=1}^{m}$ can be calculated within O(m) arithmetic operations, to calculate $u_{i},v_{i}\;i=1,2,\cdots m$, O(m) arithmetic operations are needed. Then

(16) $$\sum_{j=1}^{m}\lambda^{\left|i-j\right|}\left|i-j\right|r_{j}=u_{i}+v_{i},\;i=1,2,\cdots m,$$

can also be calculated within O(m) arithmetic operations.

Meanwhile, let

$$\zeta_{i}=\sum_{j=1}^{i}\lambda^{\left|i-j\right|}{\left|i-j\right|}^{2}r_{j},\;\eta_{i}=\sum_{j=i+1}^{m}\lambda^{\left|i-j\right|}{\left|i-j\right|}^{2}r_{j}.$$

Then, it holds that

$$\begin{array}{cc} \lambda \zeta_{i} & =\sum_{j=1}^{i}\lambda^{i-j+1}{\left(i-j\right)}^{2}r_{j}\\ \; & =\sum_{j=1}^{i}\lambda^{i-j+1}{\left(i-j+1\right)}^{2}r_{j}-2\sum_{j=1}^{i}\lambda^{i-j+1}\left(i-j\right)r_{j}-\sum_{j=1}^{i}\lambda^{i-j+1}r_{j}\\ \; & =\zeta_{i+1}-2\lambda u_{i}-\lambda b_{i}. \end{array}$$

Similarly,

$$\lambda \eta_{i}=\eta_{i-1}-\lambda r_{i}-2\lambda v_{i}-\lambda c_{i}.$$

Thus, we obtain the following recursive formulae:

$$\begin{array}{cc} \; & \zeta_{i+1}=\lambda \left(\zeta_{i}+2u_{i}+b_{i}\right),\;i=1,2\cdots m-1,\\ \; & \eta_{i-1}=\lambda \left(\eta_{i}+2v_{i}+c_{i}+r_{i}\right),\;i=m,m-1\cdots 2, \end{array}$$

with initial values $\zeta_{1}=0,\;\eta_{m}=0$. In each recursion, we perform five additions and four multiplications. Hence, to calculate $\zeta_{i},\eta_{i}\;i=1,2,\cdots m$, O(m) arithmetic operations are needed. Then

$$\sum_{j=1}^{m}\lambda^{\left|i-j\right|}{\left|i-j\right|}^{2}r_{j}=\zeta_{i}+\eta_{i},\;i=1,2,\cdots m,$$

can also be calculated within O(m) arithmetic operations.

Since $\sum_{j=1}^{m}\lambda^{\left|i-j\right|}r_{j},\sum_{j=1}^{m}\lambda^{\left|i-j\right|}\left|i-j\right|r_{j},\sum_{j=1}^{m}\lambda^{\left|i-j\right|}{\left|i-j\right|}^{2}r_{j},\;i=1,2,\cdots m$ can be calculated within O(m) arithmetic operations, we can use only O(m) arithmetic operations to calculate $G^{\prime }\left(\beta \right)$. Similarly, G(β) can also be calculated within O(m) arithmetic operations. Thus, each Newton’s iteration involves O(m) arithmetic operations. Due to the locally quadratic convergence of the Newton’s method, we need log|logε| iterations to achieve ε-accuracy. Hence, we only need O(mlog|logε|) arithmetic operations to implement (14a). Notably, the iteration (14b) can be divided into two steps:

(17) $$\begin{array}{cc} \; & a_{i}\leftarrow \sum_{j=1}^{m}\lambda^{\left|i-j\right|}r_{j},\;i=1,2\cdots m,\;r_{j}\leftarrow r_{j}\sum_{i=1}^{m}\frac{p_{i}\lambda^{\left|i-j\right|}}{a_{i}},\;j=1,2\cdots m. \end{array}$$

Based on the above analysis, each iteration of (17) can be carried out with linear computational complexity O(m).

Therefore, in each iteration of (14), it involves $O\left(m(1+\log|\log\varepsilon |)\right)$ arithmetic operations. Due to [12] (Theorem 3.2), we need O(logm/ε) iterations. Thus, the total computational cost of our method is $O\left(\frac{m\log m}{\varepsilon }\left(1+\log|\log\varepsilon |\right)\right)$. Since m∼1/ε to ensure the accuracy of numerical integration, the computational cost turns out to be $O\left(\frac{\left|\log\varepsilon \right|}{\varepsilon^{2}}\left(1+\log|\log\varepsilon |\right)\right)$ to achieve ε-accuracy. Meanwhile, the space cost is O(m), since only O(m) variables are stored. The corresponding pseudo-code is shown in Algorithm 2.
**Algorithm 2** Accelerated CBA algorithm for absolute-error distortion (1D)**Input:** 
Source distribution $p_{i}$, target distortion *D*, maximum number of iterations max_iter**Output:** 
Minimal value $\sum_{i=1}^{m}\sum_{j=1}^{m}p_{i}w_{ij}\log\frac{w_{ij}}{r_{j}}$  1:**Initialization:** $r=1_{m}/m,\beta =0$  2:**for** ℓ=1:max_iter **do**  3:      **while** |G(β)|≥ε **do**  4:            $\lambda \leftarrow e^{-\beta h}$  5:            $b_{1}\leftarrow r_{1},\;c_{m}\leftarrow 0,\;u_{1}\leftarrow 0,\;v_{m}\leftarrow 0,\;\zeta_{1}\leftarrow 0,\;\eta_{m}\leftarrow 0$  6:            **for** i=1:m−1 **do**  7:                  $b_{i+1}\leftarrow \lambda b_{i}+r_{i+1}$  8:                  $c_{m-i}\leftarrow \lambda \left(c_{m-i+1}+r_{m-i+1}\right)$  9:                  $u_{i+1}\leftarrow \lambda \left(u_{i}+b_{i}\right)$10:                  $v_{m-i}\leftarrow \lambda \left(v_{m-i+1}+c_{m-i+1}+r_{m-i+1}\right)$11:                  $\zeta_{i+1}\leftarrow \lambda \left(\zeta_{i}+2u_{i}+b_{i}\right)$12:                  $\eta_{m-i}\leftarrow \lambda \left(\eta_{m-i+1}+2v_{m-i+1}+c_{m-i+1}+r_{m-i+1}\right)$13:            $\beta \leftarrow \beta -\left(\sum_{i=1}^{m}p_{i}h\frac{\left(u_{i}+v_{i}\right)}{\left(b_{i}+c_{i}\right)}-D\right)/\left(\sum_{i=1}^{m}p_{i}h^{2}\frac{-\left(\zeta_{i}+\eta_{i}\right)\left(b_{i}+c_{i}\right)+{\left(u_{i}+v_{i}\right)}^{2}}{{\left(b_{i}+c_{i}\right)}^{2}}\right)$14:      $a_{i}\leftarrow b_{i}+c_{i}$15:      $b_{1}^{\prime }\leftarrow p_{1}/a_{1},\;c_{m}^{\prime }\leftarrow 0$16:      **for** j=1:m−1 **do**17:            $b_{j+1}^{\prime }\leftarrow \lambda b_{j}^{\prime }+p_{j+1}/a_{j+1}$18:            $c_{m-j}^{\prime }\leftarrow \lambda \left(c_{m-j+1}^{\prime }+p_{m-j+1}/a_{m-j+1}\right)$19:      $r_{j}\leftarrow r_{j}\left(b_{j}^{\prime }+c_{j}^{\prime }\right)$

After obtaining the optimal variables β and r via iterations (14), we can calculate the rate *R* for given target distortion *D* directly:

(18) $$\begin{array}{cc} R=I(X;Y) & =\sum_{i=1}^{m}\sum_{j=1}^{m}p_{i}w_{ij}\log\frac{w_{ij}}{r_{j}}=\sum_{i=1}^{m}\sum_{j=1}^{m}p_{i}w_{ij}\log\frac{e^{-\beta \rho_{ij}}}{\sum_{j=1}^{m}e^{-\beta \rho_{ij}}r_{j}}\\ \; & =-\beta \sum_{i=1}^{m}\sum_{j=1}^{m}p_{i}w_{ij}\rho_{ij}-\sum_{i=1}^{m}p_{i}\log\sum_{j=1}^{m}e^{-\beta \rho_{ij}}r_{j}=-\beta D-\sum_{i=1}^{m}p_{i}\log\sum_{j=1}^{m}\lambda^{\left|i-j\right|}r_{j}. \end{array}$$

Here, we have used the condition $\sum_{j}w_{ij}=1$. Since $\sum_{j=1}^{m}\lambda^{\left|i-j\right|}r_{j},\;i=1,2,\cdots m$ can be calculated within O(m) arithmetic operations, we only need O(m) arithmetic operations to obtain *R*.

**Example** **2.** *We evaluate our accelerated algorithms on one-dimensional continuous sources and compare them with the baseline of the CBA algorithm without acceleration. We consider two classical examples—Gaussian and Laplacian sources—and evaluate each source under absolute-error distortion measures. We set the accuracy to ε=0.001, yielding m=n=1000 discretization nodes for X and Y, and we cap the number of iterations at max_iter=1000. The discretization nodes are uniformly sampled from the interval [−10,10].*

*Under the absolute-error distortion setting, the computational time and the discrepancy in the output rate R between the two algorithms for different values of β are reported in Table 2. As shown in the table, We can see that the Fast CBA algorithm has an overwhelming advantage in computational speed. Moreover, the rate R obtained by the two algorithms are almost identical.*

**Figure 3:**
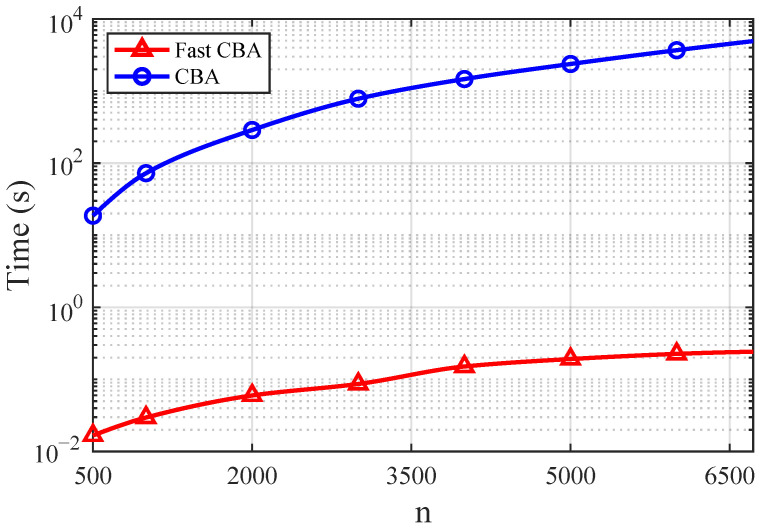
The comparison of computational time between the Fast CBA algorithm and the CBA algorithm with different numbers of grid points *n* under absolute-error distortion.

*Fixing D=0.4, by data fitting, we can see that the empirical complexity of the Fast CBA algorithm is $O(n^{1.07})$, while that of the CBA algorithm is $O(n^{2.13})$; see Figure 3 for illustration.*

#### 4.2.2. Extension to Higher Dimensions

Next, we consider the case $X=Y=R^{d}$ with the distortion measure $\rho \left(x,y\right)={∥x-y∥}_{1}=\sum_{i=1}^{d}\left|x^{i}-y^{i}\right|$, where $x=\left(x^{1},x^{2},\cdots ,x^{d}\right),\;y=\left(y^{1},y^{2},\cdots ,y^{d}\right)\in R^{d}$. Thus, after discretization, $\rho_{ij}=\sum_{k=1}^{d}|x_{i_{k}}^{k}-y_{j_{k}}^{k}|=\sum_{k=1}^{d}\left|i_{k}-j_{k}\right|h,\;i_{1},i_{2},\cdots ,i_{d},j_{1},j_{2},\cdots j_{d}=1,2,\cdots M$ and $i=\left(i_{1},i_{2},\cdots ,i_{d}\right),j=\left(j_{1},j_{2},\cdots ,j_{d}\right)$. The total number of discretization nodes is $m=M^{d}$. Here, we adopt the uniform discretization with step size *h*. For simplicity, let $\lambda =e^{-\beta h}$, $\left|i-j\right|=\sum_{k=1}^{d}\left|i_{k}-j_{k}\right|$, and $\sum_{i}$ be $\sum_{i_{1},i_{2}\cdots ,i_{d}=1}^{M}$, i.e., taking the summation over all indices $i_{1},i_{2},\cdots ,i_{d}$. Then, $e^{-\beta \rho_{ij}}=\prod_{k=1}^{d}\lambda^{|i_{k}-j_{k}|}=\lambda^{\left|i-j\right|}$.

Similarly to the one-dimensional case, we only need to accelerate the calculation of (14a) and (14b) with dimension *d*. For (14b),

$$\begin{array}{cc} \sum_{j}\lambda^{\left|i-j\right|}r_{j} & =\sum_{j_{1},\cdots j_{d}=1}^{M}\lambda^{|i_{1}-j_{1}|}\cdots \lambda^{|i_{d}-j_{d}|}r_{j_{1}\cdots j_{d}}\\ \; & =\sum_{j_{1},\cdots j_{d-1}=1}^{M}\lambda^{|i_{1}-j_{1}|}\cdots \lambda^{|i_{d-1}-j_{d-1}|}\sum_{j_{d}=1}^{M}\lambda^{|i_{d}-j_{d}|}r_{j_{1}\cdots j_{d}}. \end{array}$$

By fixing $j_{1},\cdots ,j_{d-1}$, we need O(M) arithmetic operations to calculate ${\tilde{r}}_{i_{d},j_{1}\cdots j_{d-1}}=\sum_{j_{d}=1}^{M}\lambda^{|i_{d}-j_{d}|}r_{j_{1}\cdots j_{d}},\;i_{d}=1,2\cdots M$. Thus, ${\tilde{r}}_{i_{d},j_{1}\cdots j_{d-1}},\;i_{d},j_{1},\cdots j_{d-1}=1,2\cdots M$ can be calculated within $O\left(M^{d}\right)=O\left(m\right)$ arithmetic operations. Then,

$$\begin{array}{c} \sum_{j}\lambda^{\left|i-j\right|}r_{j}=\sum_{j_{1},\cdots j_{d-1}=1}^{M}\lambda^{|i_{1}-j_{1}|}\cdots \lambda^{|i_{d-1}-j_{d-1}|}{\tilde{r}}_{i_{d},j_{1}\cdots j_{d-1}},\;i_{1},\cdots i_{d-1}=1,2\cdots M, \end{array}$$

which is exactly the case for dimension d−1 when fixing $i_{d}$. Set T(k) as the complexity to calculate $\sum_{j}\lambda^{\left|i-j\right|}r_{j}$ for dimension *k*. Then, the recursive formula for T(k) becomes:

$$T\left(k\right)=O\left(M^{k}\right)+M\cdot T\left(k-1\right).$$

Since T(1) is the complexity of calculating $\sum_{j=1}^{M}\lambda^{\left|i-j\right|}r_{j}$, we have T(1)=O(M). Therefore, T(d)=O(Md)=O(m).

For (14a), the main arithmetic complexity lies in the calculation of $\sum_{j}\lambda^{\left|i-j\right|}\left|i-j\right|r_{j}$ and $\sum_{j}\lambda^{\left|i-j\right|}{\left|i-j\right|}^{2}r_{j}$.

Note that

$$\begin{array}{cc} \sum_{j}\lambda^{\left|i-j\right|}\left|i-j\right|r_{j} & =\sum_{k=1}^{d}\sum_{j_{1},\cdots j_{d}=1}^{M}\lambda^{|i_{1}-j_{1}|}\cdots \lambda^{|i_{d}-j_{d}|}\left|i_{k}-j_{k}\right|r_{j_{1}\cdots j_{d}}\\ \; & =\sum_{k=1}^{d}\;\sum_{j_{1}\cdots j_{k-1},j_{k+1}\cdots j_{d}=1}^{M}\;\prod_{s\neq k}\lambda^{|i_{s}-j_{s}|}\sum_{j_{k}=1}^{M}\lambda^{|i_{k}-j_{k}|}\left|i_{k}-j_{k}\right|r_{j_{1}\cdots j_{d}}. \end{array}$$

Fixing $j_{1}\cdots j_{k-1},j_{k+1}\cdots j_{d}$, we can then calculate ${\tilde{r}}_{i_{k},j_{1}\cdots j_{k-1},j_{k+1}\cdots j_{d}}=\sum_{j_{k}=1}^{M}\lambda^{|i_{k}-j_{k}|}\left|i_{k}-j_{k}\right|r_{j_{1}\cdots j_{d}},\;i_{k}=1,2\cdots M$ within O(M) arithmetic operations. Furthermore, ${\tilde{r}}_{i_{k},j_{1}\cdots j_{k-1},j_{k+1}\cdots j_{d}}$, $i_{k},j_{1}\cdots j_{k-1},j_{k+1}\cdots j_{d}=1,2\cdots M$ can be calculated within $O\left(M^{d}\right)=O\left(m\right)$ arithmetic operations. Meanwhile, $\sum_{j_{1}\cdots j_{k-1},j_{k+1}\cdots j_{d}=1}^{M}\prod_{s\neq k}\lambda^{|i_{s}-j_{s}|}{\tilde{r}}_{i_{k},j_{1}\cdots j_{k-1},j_{k+1}\cdots j_{d}}$ can be calculated within O(m) arithmetic operations due to the aforementioned analysis. Thus, O(dm)=O(m) arithmetic operations are needed to calculate $\sum_{j}\lambda^{\left|i-j\right|}\left|i-j\right|r_{j}$.

In the meantime,

$$\begin{array}{cc} \; & \sum_{j}\lambda^{\left|i-j\right|}{\left|i-j\right|}^{2}r_{j}=\sum_{k,\ell =1}^{d}\sum_{j_{1},\cdots j_{d}=1}^{M}\lambda^{\left|i_{1}-j_{1}\right|}\cdots \lambda^{|i_{d}-j_{d}|}\left|i_{k}-j_{k}\right|\left|i_{\ell }-j_{\ell }\right|r_{j_{1}\cdots j_{d}}\\ \; & =\sum_{k\neq \ell }\;\sum_{j_{1}\cdots j_{k-1},j_{k+1}\cdots j_{\ell -1},j_{\ell +1}\cdots j_{d}=1}^{M}\;\prod_{s\neq k,\ell }\lambda^{\left|i_{s}-j_{s}\right|}\sum_{j_{\ell }=1}^{M}\lambda^{\left|i_{\ell }-j_{\ell }\right|}\left|i_{\ell }-j_{\ell }\right|\sum_{j_{k}=1}^{M}\lambda^{\left|i_{k}-j_{k}\right|}\left|i_{k}-j_{k}\right|r_{j_{1}\cdots j_{d}}\\ \; & \;+\sum_{k=1}^{d}\;\sum_{j_{1}\cdots j_{k-1},j_{k+1}\cdots j_{d}=1}^{M}\;\prod_{s\neq k}\lambda^{|i_{s}-j_{s}|}\sum_{j_{k}=1}^{M}\lambda^{|i_{k}-j_{k}|}{\left|i_{k}-j_{k}\right|}^{2}r_{j_{1}\cdots j_{d}}. \end{array}$$

By similarly fixing $j_{1},\cdots ,j_{k-1},j_{k+1},\cdots ,j_{d}$, each component of $\sum_{j}\lambda^{\left|i-j\right|}{\left|i-j\right|}^{2}r_{j}$ can be computed with O(m) arithmetic operations. Therefore, we obtain the estimate of computational complexity $O\left(\frac{\left|\log\varepsilon \right|}{\varepsilon^{d+1}}\left(1+\log|\log\varepsilon |\right)\right)$ to achieve ε-accuracy of our method. Moreover, the rate *R* can also be calculated using $O\left(m\right)=O\left(1/\varepsilon^{d}\right)$ arithmetic operations.

## 5. Conclusions

In this article, we conducted an integrated approach for computing rate–distortion functions, connecting the RD problem formulation of continuous sources and discretization schemes along with their corresponding efficient discrete algorithms. We first showed that discretized reproduction distributions provably converge to the solution of the underlying continuous RD problem, and derived quantitative error bounds that clarify the impact of discretization resolution and problem dimension. In the discrete setting, we revisited the Blahut–Arimoto (BA) and constrained BA (CBA) algorithms, establishing their arithmetic complexity properties for achieving ϵ-accuracy guarantees when used, together with discretization, as solvers for the underlying continuous RD problem. For certain distortion measures, we further designed acceleration techniques that exploit the structure in discretized problems, including squared-error and absolute-error distortion measures.

## Figures and Tables

**Table 1:** Comparison between FFT-based Fast CBA and CBA algorithms under squared-error distortion.

Source	(*D*, *R*, Slope β)	Time (s)		Ratio	Error
		$t_{\mathrm{Fast}}$	$t_{\mathrm{CB}A}$	**Speed-Up**	$|R_{\mathrm{CBA}}-R_{\mathrm{Fast}}|$
Gaussian	(0.1,1.1513,5.0000)	0.6274	27.7583	44.2417	$9.7700\times 10^{-15}$
	(0.2,0.8042,2.5000)	0.6291	30.1018	47.8485	$9.1938\times 10^{-15}$
	(0.3,0.6020,1.6667)	0.6528	30.9772	47.4542	$9.4369\times 10^{-15}$
	(0.4,0.4581,1.2500)	0.6600	31.1232	47.1533	$3.6082\times 10^{-15}$
Laplacian	(0.1,0.7343,4.9682)	1.5500	51.9308	33.5044	$5.8176\times 10^{-14}$
	(0.2,0.3932,2.4218)	1.5995	56.6702	35.4304	$4.2355\times 10^{-14}$
	(0.3,0.2014,1.5225)	1.7628	57.2084	32.4523	$8.5765\times 10^{-15}$
	(0.4,0.0766,1.0040)	1.5863	56.7330	35.7645	$3.1346\times 10^{-15}$

Notes: We take σ=1 for Gaussian source $p\left(x\right)=\frac{1}{\sqrt{2\pi \sigma^{2}}}e^{-\frac{x^{2}}{2\sigma^{2}}}$ and b=0.5 for Laplacian source $p\left(x\right)=\frac{1}{2b}e^{-\frac{\left|x\right|}{b}}$.

**Table 2:** Comparison between Fast CBA and CBA algorithms under absolute-error distortion.

Source	(*D*, *R*, Slope β)	Time (s)		Ratio	Error
		$t_{\mathrm{Fast}}$	$t_{\mathrm{CB}A}$	**Speed-Up**	$|R_{\mathrm{CBA}}-R_{\mathrm{Fast}}|$
Gaussian	(0.1,2.0251,9.9345)	0.0394	74.7331	$1.8945\times 10^{3}$	$8.7953\times 10^{-11}$
	(0.2,1.3344,4.9917)	0.0425	80.5380	$1.8961\times 10^{3}$	$1.2735\times 10^{-10}$
	(0.3,0.9294,3.3303)	0.0771	138.1042	$1.7916\times 10^{3}$	$1.3441\times 10^{-9}$
	(0.4,0.6422,2.4901)	0.0752	140.3673	$1.8667\times 10^{3}$	$1.9662\times 10^{-8}$
Laplacian	(0.1,1.6062,9.9345)	0.0761	156.8812	$2.0616\times 10^{3}$	$7.6961\times 10^{-9}$
	(0.2,0.9155,4.9916)	0.0748	151.2206	$2.0210\times 10^{3}$	$4.9796\times 10^{-8}$
	(0.3,0.5106,3.3307)	0.0762	148.8361	$1.9539\times 10^{3}$	$1.2569\times 10^{-7}$
	(0.4,0.2231,2.4986)	0.0767	150.7649	$1.9655\times 10^{3}$	$2.2397\times 10^{-7}$

Notes: We take σ=1 for Gaussian source $p\left(x\right)=\frac{1}{\sqrt{2\pi \sigma^{2}}}e^{-\frac{x^{2}}{2\sigma^{2}}}$ and b=0.5 for Laplacian source $p\left(x\right)=\frac{1}{2b}e^{-\frac{\left|x\right|}{b}}$.

## Data Availability

All data generated for this study are presented within the paper. Scripts and numerical data can be shared by the corresponding author upon reasonable request.

## Appendix A. Proofs in Section 2

### Appendix A.1. Proof of Lemma 1

**Proof.** We denote the optimal solution of (5) as $r^{*}$ and $W_{n}=\left\{\sum_{j=1}^{n}r_{j}\delta_{y_{j}^{n}}|r_{j}\geq 0,\sum_{j=1}^{n}r_{j}=1\right\}$. As shown in Section 2.1, the continuous RD problem has the discrete form (6), which is in fact minimizing f(r) as defined in (5), but over $W_{n}$ instead of W. Denote the optimal solution of the discrete problem as $r^{n}$. Then $f\left(r^{n}\right)\leq f\left(r\right),\forall r\in W_{n}$. Notice that if there exists ${\tilde{q}}^{n}\in W_{n}$ satisfying $f\left({\tilde{q}}^{n}\right)\to f\left(r^{*}\right)$, then

(A1) $$f\left(r^{*}\right)\leq f\left(r^{n}\right)\leq f\left({\tilde{q}}^{n}\right)\to f\left(r^{*}\right).$$

Here, the first inequality is due to the fact $W_{n}\subseteq W$. The relationship (A1) would then lead to the convergence $f\left(r^{n}\right)\to f\left(r^{*}\right)$. Now, let $q^{n}=\sum_{j=1}^{n}A_{j}^{n}\delta_{y_{j}^{n}}$, where $A_{j}^{n}=\int_{I_{j}}dr^{*}$ and $I_{j}$ is defined in Section 2.1. Let ${\tilde{q}}^{n}=q^{n}/\int dq^{n}$. Since $\int dq^{n}=\sum_{j}\int_{I_{j}}dr^{*}\leq 1$, one has

$$f\left({\tilde{q}}^{n}\right)=f\left(q^{n}\right)+\log\left(\int dq^{n}\right)\leq f\left(q^{n}\right).$$

Since ${\tilde{q}}^{n}\in W_{n}$ and $f\left({\tilde{q}}^{n}\right)\leq f\left(q^{n}\right)$, it suffices to show that $f\left(q^{n}\right)\to f\left(r^{*}\right)$. Before doing so, convergence under a truncation of X needs to be established. Choose A>1 such that $\int_{∥y∥\leq A-1}dr^{*}\left(y\right)\geq \frac{1}{2}$, and fix any M≥A. First, for $x\in {\left[-M,M\right]}^{d}$ and sufficiently large *n*, the following estimate holds:

$$\begin{array}{cc} \; & \left|\int \exp\left(-\beta \rho \left(x,y\right)\right)dq^{n}\left(y\right)-\int \exp\left(-\beta \rho \left(x,y\right)\right)dr^{*}\left(y\right)\right|\\ \; & \leq \sum_{i}\int_{I_{i}}\left|\exp\left(-\beta \rho \left(x,y\right)\right)-\exp\left(-\beta \rho \left(x,y_{i}^{n}\right)\right)\right|dr^{*}\left(y\right)+\int_{|y|>n^{\frac{1}{2d}}}\exp\left(-\beta \rho \left(x,y\right)\right)dr^{*}\left(y\right)\\ \; & \leq \sum_{i}\int_{I_{i}}\varepsilon dr^{*}\left(y\right)+\int_{|y|>n^{\frac{1}{2d}}}dr^{*}\left(y\right)\leq 2\varepsilon , \end{array}$$

where the second inequality invokes the assumption together with the decomposition $q^{n}=\sum_{j}\int_{I_{j}}dr^{*}\;\delta_{y_{j}^{n}}$. Next, we establish the uniform convergence. For $x\in {\left[-M,M\right]}^{d}$,

(A2) $$\begin{array}{cc} \; & \int \exp\left(-\beta \rho \left(x,y\right)\right)dq^{n}\left(y\right)\geq \int_{∥y∥\leq M}\exp\left(-\beta \rho \left(x,y\right)\right)dq^{n}\left(y\right)\\ \; & \geq \int_{∥y∥\leq M}\exp\left(-\beta \rho^{*}\right)dq^{n}\left(y\right)\geq \frac{1}{2}\exp\left(-\beta \rho^{*}\right)≜\delta . \end{array}$$

Here $\rho^{*}=\max_{x,y\in {\left[-M,M\right]}^{d}}\rho \left(x,y\right)$ and $\int_{∥y∥\leq M}dq^{n}\geq \frac{1}{2}$ is due to

(A3) $$\begin{array}{cc} \int_{∥y∥\leq A}dq^{n}\left(y\right) & =\sum_{i:y_{i}^{n}\in {\left[-A,A\right]}^{d}}\int_{I_{i}}dr^{*}\left(y\right)\geq \int_{∥y∥\leq A-1}dr^{*}\left(y\right)\geq \frac{1}{2}. \end{array}$$

Since logx is uniformly continuous over [δ,+∞),

$$\begin{array}{c} \log\left(\int \exp\left(-\beta \rho \left(x,y\right)\right)dq^{n}\left(y\right)\right)⇉\log\left(\int \exp\left(-\beta \rho \left(x,y\right)\right)dr^{*}\left(y\right)\right),x\in {\left[-M,M\right]}^{d}. \end{array}$$

Then, for sufficiently large *n*,

$$\begin{array}{cc} \; & |\int_{∥x∥\leq M}\log\left[\int \exp\left(-\beta \rho \left(x,y\right)\right)dq^{n}\left(y\right)\right]dp\left(x\right)\\ \; & \;-\int_{∥x∥\leq M}\log\left[\int \exp\left(-\beta \rho \left(x,y\right)\right)dr^{*}\left(y\right)\right]dp\left(x\right)|\leq \int_{∥x∥\leq M}\varepsilon dp\left(x\right)\leq \varepsilon . \end{array}$$

As n→+∞,

$$\begin{array}{c} \int_{∥x∥\leq M}\log\left[\int \exp\left(-\beta \rho \left(x,y\right)\right)dq^{n}\left(y\right)\right]dp\left(x\right)\to \\ \;\;\;\;\;\;\;\;\;\;\;\;\;\;\;\;\;\;\;\;\;\;\;\;\;\;\;\;\;\;\;\;\;\;\;\;\;\;\;\;\;\;\;\;\;\;\;\;\;\;\;\;\;\;\;\;\;\;\;\;\;\;\;\;\;\;\;\;\int_{∥x∥\leq M}\log\left[\int \exp\left(-\beta \rho \left(x,y\right)\right)dr^{*}\left(y\right)\right]dp\left(x\right). \end{array}$$

Hence, convergence after truncation holds. It remains to prove that $f\left(q^{n}\right)\to f\left(r^{*}\right)$. For M≥A,

(A4) $$\begin{array}{cc} \; & |f\left(q^{n}\right)-f\left(r^{*}\right)|=|\int H_{q^{n}\left(y\right)}\left(x\right)dp\left(x\right)-\int H_{r^{*}\left(y\right)}\left(x\right)dp\left(x\right)|\\ \; & \leq |\int_{∥x∥\leq M}\left[H_{q^{n}\left(y\right)}\left(x\right)-H_{r^{*}\left(y\right)}\left(x\right)\right]dp\left(x\right)|+\int_{∥x∥>M}\left(-H_{q^{n}\left(y\right)}\left(x\right)-H_{r^{*}\left(y\right)}\left(x\right)\right)dp\left(x\right), \end{array}$$

where $H_{r(y)}\left(x\right)=\log\int \exp\left(-\beta \rho \left(x,y\right)\right)dr\left(y\right)$. Let $g\left(x\right)=\max_{∥y∥\leq A}\rho \left(x,y\right)$. Note that

$$\begin{array}{cc} \int \exp\left(-\beta \rho \left(x,y\right)\right)dq^{n}\left(y\right) & \geq \exp\left(-\beta g\left(x\right)\right)\int_{∥y∥\leq A}dq^{n}\left(y\right)\geq \frac{1}{2}\exp\left(-\beta g\left(x\right)\right), \end{array}$$

where the second inequality is similar to (A3). Similarly, we have

$$\int \exp\left(-\beta \rho \left(x,y\right)\right)dr^{*}\left(y\right)\geq \frac{1}{2}\exp\left(-\beta g\left(x\right)\right).$$

Thus, combined with the assumption, we get

$$\begin{array}{cc} \; & \int_{∥x∥>M}\left(-H_{q^{n}}\left(x\right)-H_{r^{*}}\left(x\right)\right)dp\left(x\right)\leq \int_{∥x∥>M}\;\;\;\left(2\log2+2\beta g\left(x\right)\right)dp\left(x\right)\to 0,\;M\to +\infty . \end{array}$$

Therefore, by (A4) in the proof of Lemma 2,

$$\begin{array}{cc} |f\left(q^{n}\right)-f\left(r^{*}\right)|\; & \leq |\int_{∥x∥\leq M}\;\;\;\;\left[H_{q^{n}\left(y\right)}\left(x\right)-H_{r^{*}\left(y\right)}\left(x\right)\right]dp\left(x\right)|\;+\;\int_{∥x∥>M}\;\;\;\;\;\left(2\log2+2\beta g\left(x\right)\right)dp\left(x\right). \end{array}$$

By taking the upper limit of *n* for this inequality, we obtain

$$\begin{array}{cc} \; & \underset{n}{\lim\;\sup}|f\left(q^{n}\right)-f\left(r^{*}\right)|\leq \int_{∥x∥>M}\left(2\log2+2\beta g\left(x\right)\right)dp\left(x\right). \end{array}$$

Taking the limit M→+∞, it follows that $f\left(q^{n}\right)\to f\left(r^{*}\right)$. To prove the convergence of the solutions, let $\tilde{r}$ be a limit point of the solution sequence ${\left\{r^{n}\right\}}_{n=1}^{\infty }$. Then there exists a subsequence ${\left\{r^{n_{k}}\right\}}_{k=1}^{\infty }$ satisfying $r^{n_{k}}\overset{w}{\to }\tilde{r}$. Next,

$$f\left(\tilde{r}\right)=\lim_{k}f\left(r^{n_{k}}\right)=f\left(r^{*}\right),$$

since $\lim_{n}f\left(r^{n}\right)=f\left(r^{*}\right)$. Therefore, we have shown that $f(\tilde{r})$ is equal to the optimal value, and consequently $\tilde{r}$ is an optimal solution. □

### Appendix A.2. Proof of Proposition 1

**Proof.** Denote $W_{n}=\left\{\sum_{j=1}^{n}r_{j}\delta_{y_{j}^{n}}|r_{j}\geq 0,\sum_{j=1}^{n}r_{j}=1\right\}$. Similarly to Lemma 1, the discrete problem (7) is equivalent to optimizing F(r,β) over $W_{n}$. By the property of max–min problems (7) and (3), $\forall r\in W_{n},\forall \beta \geq 0$,

$$F\left(r^{n},\beta^{n}\right)\leq F\left(r,\beta^{n}\right),\;F\left(r^{n},\beta^{n}\right)\geq F\left(r^{n},\beta \right).$$

Furthermore, ∀r∈W,∀β≥0,

$$F\left(r^{*},\beta^{*}\right)\leq F\left(r,\beta^{*}\right),\;F\left(r^{*},\beta^{*}\right)\geq F\left(r^{*},\beta \right).$$

Next, let ${\tilde{q}}^{n}=q^{n}/\int dq^{n}\in W_{n}$. Since $\int dq^{n}\leq 1$,

$$F\left({\tilde{q}}^{n},\beta \right)=F\left(q^{n},\beta \right)+\log\left(\int dq^{n}\right)\leq F\left(q^{n},\beta \right),\;\forall \beta \geq 0.$$

Consequently, the following chain of inequalities holds:

$$F\left(r^{*},\beta^{*}\right)\leq F\left(r^{n},\beta^{*}\right)\leq F\left(r^{n},\beta^{n}\right)\leq F\left({\tilde{q}}^{n},\beta^{n}\right)\leq F\left(q^{n},\beta^{n}\right)\leq F\left(q^{n},{\tilde{\beta }}^{n}\right),$$

where ${\tilde{\beta }}^{n}={\mathrm{argmax}}_{\beta }F\left(q^{n},\beta \right)$ and we have used the max–min optimality property of $\left(r^{*},\beta^{*}\right)$. Note that if $F\left(q^{n},{\tilde{\beta }}^{n}\right)\to F\left(r^{*},\beta^{*}\right)$, then

(A5) $$F\left(r^{*},\beta^{*}\right)\leq F\left(r^{n},\beta^{n}\right)\leq F\left(q^{n},{\tilde{\beta }}^{n}\right)\to F\left(r^{*},\beta^{*}\right).$$

Therefore, we obtain the convergence $F\left(r^{n},\beta^{n}\right)\to F\left(r^{*},\beta^{*}\right)$. In summary, we only need to prove $F\left(q^{n},{\tilde{\beta }}^{n}\right)\to F\left(r^{*},\beta^{*}\right)$. □

### Appendix A.3. Proof of Lemma 2

**Proof.** (1). We first show that the sequence $\left\{{\tilde{\beta }}^{\;n}\right\}$ is bounded away from zero, i.e.,

$${\tilde{\beta }}^{\;n}\geq B_{1}>0,\;\forall n.$$

Suppose, to the contrary, that there exists a subsequence with ${\tilde{\beta }}^{\;n_{k}}\to 0$. Therefore, there exists $k_{0}$ such that for all $k\geq k_{0}$, ${\tilde{\beta }}^{n_{k}}<\beta^{*}/2$. Here we assume $\beta^{*}>0$ and the degenerate case $\beta^{*}=0$ can be disregarded. By optimality, ${\tilde{\beta }}^{\;n}$ satisfies the first-order optimality condition and thus is a root of a monotone function

$$G_{q^{n}}\left(\beta \right)=\int \frac{\left(\int e^{-\beta \rho (x,y)}\rho \left(x,y\right)dq^{n}\left(y\right)\right)}{\left(\int e^{-\beta \rho (x,y)}dq^{n}\left(y\right)\right)}dp\left(x\right)-D.$$

Then by the monotone property of $G_{q^{n_{k}}}$, we have

$$0=G_{q^{n_{k}}}\left({\tilde{\beta }}^{n_{k}}\right)>G_{q^{n_{k}}}\left(\beta^{*}/2\right).$$

The monotonicity follows from $G_{q^{\;n_{k}}}^{\prime }\left(\beta \right)<0$, which is ensured by the Cauchy–Schwarz inequality. Thus, for sufficiently large *M*,

$$\begin{array}{cc} D & >\int \frac{e^{-\beta^{*}\rho \left(x,y\right)/2}\rho \left(x,y\right)dq^{n_{k}}\left(y\right)}{\int e^{-\beta^{*}\rho \left(x,y\right)/2}dq^{n_{k}}\left(y\right)}dp\left(x\right)\geq \int_{∥x∥\leq M}\frac{\int e^{-\beta^{*}\rho \left(x,y\right)/2}\rho \left(x,y\right)dq^{n_{k}}\left(y\right)}{\int e^{-\beta^{*}\rho \left(x,y\right)/2}dq^{n_{k}}\left(y\right)}dp\left(x\right). \end{array}$$

By the proof of Lemma 1, we have

$$\int e^{-\beta^{*}\rho \left(x,y\right)/2}dq^{n_{k}}\left(y\right)⇉\int e^{-\beta^{*}\rho \left(x,y\right)/2}dr^{*}\left(y\right).$$

Similarly,

$$\int e^{-\beta^{*}\rho \left(x,y\right)/2}\rho \left(x,y\right)dq^{n_{k}}\left(y\right)\;\;⇉\;\;\int e^{-\beta^{*}\rho \left(x,y\right)/2}\rho \left(x,y\right)dr^{*}\left(y\right).$$

Similarly to (A2) in the proof of Lemma 1, we have

$$\int e^{-\beta^{*}\rho \left(x,y\right)/2}dq^{n_{k}}\left(y\right)\geq \delta_{0}.$$

Meanwhile, the upper bound can be estimated by

$$\int e^{-\beta^{*}\rho \left(x,y\right)/2}\rho \left(x,y\right)dq^{n_{k}}\left(y\right)\leq \int B\;dq^{n_{k}}\left(y\right)\leq B.$$

Here, *B* is a bound of the function $e^{-\beta^{*}x/2}x,x\geq 0$. Next, since g(x,y)=x/y is uniformly continuous over $\left[0,B\right]\times \left[\delta_{0},\infty \right)$, we have

$$\frac{\int \exp\left(-\beta^{*}\rho \left(x,y\right)/2\right)\rho \left(x,y\right)dq^{n_{k}}\left(y\right)}{\int \exp\left(-\beta^{*}\rho \left(x,y\right)/2\right)dq^{n_{k}}\left(y\right)}⇉\frac{\int \exp\left(-\beta^{*}\rho \left(x,y\right)/2\right)\rho \left(x,y\right)dr^{*}\left(y\right)}{\int \exp\left(-\beta^{*}\rho \left(x,y\right)/2\right)dr^{*}\left(y\right)}.$$

Thus, it holds that

(A6) $$\begin{array}{cc} D & \geq \int_{∥x∥\leq M}\;\;\;\;\;\frac{\int e^{-\beta^{*}\rho \left(x,y\right)/2}\rho \left(x,y\right)dq^{n_{k}}\left(y\right)}{\int e^{-\beta^{*}\rho \left(x,y\right)/2}dq^{n_{k}}\left(y\right)}dp\left(x\right)\to \int_{∥x∥\leq M}\;\;\;\;\;\;\frac{\int e^{-\beta^{*}\rho \left(x,y\right)/2}\rho \left(x,y\right)dr^{*}\left(y\right)}{\int e^{-\beta^{*}\rho \left(x,y\right)/2}dr^{*}\left(y\right)}dp\left(x\right). \end{array}$$

Letting M→∞ leads to

$$G_{r^{*}}\left(\beta^{*}/2\right)\leq 0=G_{r^{*}}\left(\beta^{*}\right).$$

Using the monotonicity of $G_{r^{*}}$, we have $\beta^{*}/2\geq \beta^{*}$, which is a contradiction. (2). To prove ${\tilde{\beta }}^{n}\to \beta^{*}$, we only need to show that every convergent subsequence converges to $\beta^{*}$, i.e., if a convergent subsequence ${\tilde{\beta }}^{n_{k}}\to \bar{\beta }$, then $\bar{\beta }=\beta^{*}$. Now, let ${\tilde{\beta }}^{n_{k}}$ be a convergent subsequence and ${\tilde{\beta }}^{n_{k}}\to \bar{\beta }$. Since ${\tilde{\beta }}^{n_{k}}$ is lower bounded away from zero, as shown in Lemma 2, we have $\bar{\beta }>0$. Let $A_{0}$ satisfy $e^{-\bar{\beta }t/2}t\leq \varepsilon$ and $e^{-\bar{\beta }t/2}\leq \varepsilon ,\;\forall t\geq A_{0}$. We have

$$\begin{array}{cc} \; & \left|\int e^{-{\tilde{\beta }}^{n_{k}}\rho \left(x,y\right)}\rho \left(x,y\right)dq^{n_{k}}\left(y\right)-\int e^{-\bar{\beta }\rho \left(x,y\right)}\rho \left(x,y\right)dq^{n_{k}}\left(y\right)\right|\\ \; & \leq \int \left|e^{-{\tilde{\beta }}^{n_{k}}\rho \left(x,y\right)}-e^{-\bar{\beta }\rho \left(x,y\right)}\right|\rho \left(x,y\right)dq^{n_{k}}\left(y\right)\leq \int e^{-\bar{\beta }\rho \left(x,y\right)}\left|e^{\left(\bar{\beta }-{\tilde{\beta }}^{n_{k}}\right)\rho \left(x,y\right)}-1\right|\rho \left(x,y\right)dq^{n_{k}}\left(y\right). \end{array}$$

Next, we divide the integral into two parts and estimate each of them. On one hand, when *k* is sufficiently large,

$$\begin{array}{cc} \; & \int_{y:\rho \left(x,y\right)>A_{0}}e^{-\bar{\beta }\rho \left(x,y\right)}\left|e^{\left(\bar{\beta }-{\tilde{\beta }}^{n_{k}}\right)\rho \left(x,y\right)}-1\right|\rho \left(x,y\right)dq^{n_{k}}\left(y\right)\\ \; & \leq \int_{y:\rho \left(x,y\right)>A_{0}}e^{-\bar{\beta }\rho \left(x,y\right)}e^{|\bar{\beta }-{\tilde{\beta }}^{n_{k}}|\rho \left(x,y\right)}\rho \left(x,y\right)dq^{n_{k}}\left(y\right)\\ \; & \leq \int_{y:\rho \left(x,y\right)>A_{0}}e^{-\bar{\beta }\rho \left(x,y\right)/2}\rho \left(x,y\right)dq^{n_{k}}\left(y\right)\leq \int_{y:\rho \left(x,y\right)>A_{0}}\varepsilon dq^{n_{k}}\left(y\right)\leq \varepsilon , \end{array}$$

where the second inequality is due to $|\bar{\beta }-{\tilde{\beta }}^{n_{k}}|\leq \bar{\beta }/2$ when *k* is sufficiently large; on the other hand, when *k* is sufficiently large,

$$\begin{array}{cc} \; & \int_{y:\rho \left(x,y\right)\leq A_{0}}e^{-\bar{\beta }\rho \left(x,y\right)}\left|e^{\left(\bar{\beta }-{\tilde{\beta }}^{n_{k}}\right)\rho \left(x,y\right)}-1\right|\rho \left(x,y\right)dq^{n_{k}}\left(y\right)\\ \; & \leq \int_{y:\rho \left(x,y\right)\leq A_{0}}\left|e^{\left(\bar{\beta }-{\tilde{\beta }}^{n_{k}}\right)\rho \left(x,y\right)}-1\right|A_{0}\;dq^{n_{k}}\left(y\right)\\ \; & \leq \int_{y:\rho \left(x,y\right)\leq A_{0}}\left(e^{|\bar{\beta }-{\tilde{\beta }}^{n_{k}}|\rho \left(x,y\right)}-1\right)A_{0}\;dq^{n_{k}}\left(y\right)\\ \; & \leq \int_{y:\rho \left(x,y\right)\leq A_{0}}\left(e^{|\bar{\beta }-{\tilde{\beta }}^{n_{k}}|A_{0}}-1\right)A_{0}\;dq^{n_{k}}\left(y\right)\\ \; & \leq \left(e^{|\bar{\beta }-{\tilde{\beta }}^{n_{k}}|A_{0}}-1\right)A_{0}\leq \varepsilon . \end{array}$$

Combining the two parts, we obtain

$$|\int e^{-{\tilde{\beta }}^{n_{k}}\rho \left(x,y\right)}\rho \left(x,y\right)dq^{n_{k}}\left(y\right)-\int e^{-\bar{\beta }\rho \left(x,y\right)}\rho \left(x,y\right)dq^{n_{k}}\left(y\right)|\leq 2\varepsilon ,$$

when *k* is sufficiently large. Thus,

(A7) $$\int e^{-{\tilde{\beta }}^{n_{k}}\rho \left(x,y\right)}\rho \left(x,y\right)dq^{n_{k}}\left(y\right)-\int e^{-\bar{\beta }\rho \left(x,y\right)}\rho \left(x,y\right)dq^{n_{k}}\left(y\right)⇉0,\;\forall x.$$

By the proof of Lemma 2,

$$\begin{array}{c} \int e^{-\bar{\beta }\rho \left(x,y\right)}\rho \left(x,y\right)dq^{n_{k}}\left(y\right)⇉\;\;\int e^{-\bar{\beta }\rho \left(x,y\right)}\rho \left(x,y\right)dr^{*}\left(y\right),\;\forall x. \end{array}$$

Thus,

$$\begin{array}{c} \int \;\;e^{-{\tilde{\beta }}^{n_{k}}\rho \left(x,y\right)}\rho \left(x,y\right)dq^{n_{k}}\left(y\right)⇉\;\;\int \;\;e^{-\bar{\beta }\rho \left(x,y\right)}\rho \left(x,y\right)dr^{*}\left(y\right),\;\forall x. \end{array}$$

Similarly,

$$\begin{array}{c} \int e^{-{\tilde{\beta }}^{n_{k}}\rho \left(x,y\right)}dq^{n_{k}}\left(y\right)⇉\int e^{-\bar{\beta }\rho \left(x,y\right)}dr^{*}\left(y\right),\;\forall x. \end{array}$$

Next, we prove that $G_{q^{n_{k}}}\left({\tilde{\beta }}^{n_{k}}\right)\to G_{r^{*}}\left(\bar{\beta }\right)$. Since ${\tilde{\beta }}^{\;n_{k}}\to \bar{\beta }$, for any ε>0 there exists $k_{0}$ such that for all $k\geq k_{0}$, $|{\tilde{\beta }}^{\;n_{k}}-\bar{\beta }|\leq \varepsilon$. Using the monotonicity of $G_{q^{n_{k}}}$, we obtain

(A8) $$G_{q^{n_{k}}}\left(\bar{\beta }+\varepsilon \right)\leq G_{q^{n_{k}}}\left({\tilde{\beta }}^{n_{k}}\right)\leq G_{q^{n_{k}}}\left(\bar{\beta }-\varepsilon \right).$$

In the following, we divide $G_{q^{n_{k}}}\left(\bar{\beta }+\varepsilon \right)$ into two parts and estimate each of them. Set $\beta :=\bar{\beta }+\varepsilon$ for brevity. We first estimate the part

$$\begin{array}{cc} \; & \int_{∥x∥>M}\frac{\left(\int e^{-\beta \rho (x,y)}\rho \left(x,y\right)dq^{n_{k}}\left(y\right)\right)}{\left(\int e^{-\beta \rho (x,y)}dq^{n_{k}}\left(y\right)\right)}dp\left(x\right). \end{array}$$

Denote $A_{x}=-\frac{1}{\beta }\;\log\int e^{-\beta \rho (x,y)}dq^{n_{k}}\left(y\right)$, then

$$\begin{array}{cc} \; & \int e^{-\beta \rho (x,y)}\rho \left(x,y\right)dq^{n_{k}}\left(y\right)\\ \; & \leq \int_{y:\rho \left(x,y\right)\leq A_{x}}e^{-\beta \rho (x,y)}\rho \left(x,y\right)dq^{n_{k}}\left(y\right)+\int_{y:\rho \left(x,y\right)>A_{x}}e^{-\beta \rho (x,y)}\rho \left(x,y\right)dq^{n_{k}}\left(y\right)\\ \; & \leq \int_{y:\rho \left(x,y\right)\leq A_{x}}e^{-\beta \rho (x,y)}A_{x}\;dq^{n_{k}}\left(y\right)+\int_{y:\rho \left(x,y\right)>A_{x}}e^{-\beta \max(A_{x},1/\beta )}\max\left(A_{x},1/\beta \right)dq^{n_{k}}\left(y\right)\\ \; & \leq A_{x}\int e^{-\beta \rho (x,y)}\;dq^{n_{k}}\left(y\right)+e^{-\beta \max(A_{x},1/\beta )}\max\left(A_{x},1/\beta \right)\\ \; & =A_{x}\exp\left(-\beta A_{x}\right)+e^{-\beta \max(A_{x},1/\beta )}\max\left(A_{x},1/\beta \right). \end{array}$$

Thus,

$$\begin{array}{cc} \; & \int_{∥x∥>M}\frac{\left(\int e^{-\beta \rho (x,y)}\rho \left(x,y\right)dq^{n_{k}}\left(y\right)\right)}{\left(\int e^{-\beta \rho (x,y)}dq^{n_{k}}\left(y\right)\right)}dp\left(x\right)\leq \int_{∥x∥>M}\left(A_{x}+\max\left(A_{x},1/\beta \right)\right)dp\left(x\right)\\ \; & \leq \int_{∥x∥>M}\left(2A_{x}+1/\beta \right)dp\left(x\right). \end{array}$$

Note that when *k* is sufficiently large, we have

$$\begin{array}{cc} A_{x} & =-\frac{1}{\beta }\;\log\int e^{-\beta \rho (x,y)}dq^{n_{k}}\left(y\right)\leq -\frac{1}{\beta }\;\log\int_{∥y∥\leq A}e^{-\beta \rho (x,y)}dq^{n_{k}}\left(y\right)\\ \; & \leq -\frac{1}{\beta }\;\log\int_{∥y∥\leq A}e^{-\beta g(x)}dq^{n_{k}}\left(y\right)\leq -\frac{1}{\beta }\;\log\left(e^{-\beta g(x)}/2\right)=g\left(x\right)+\left(\log2\right)/\beta , \end{array}$$

Here, *A* is the constant in Theorem 1 and $g\left(x\right)=\max_{∥y∥\leq A}\rho \left(x,y\right)$. Then,

$$\int \left(2A_{x}+1/\beta \right)dp\left(x\right)<\infty .$$

Similarly to (A6), the other part satisfies

$$\begin{array}{cc} \; & \int_{∥x∥\leq M}\frac{\left(\int e^{-\beta \rho (x,y)}\rho \left(x,y\right)dq^{n_{k}}\left(y\right)\right)}{\left(\int e^{-\beta \rho (x,y)}dq^{n_{k}}\left(y\right)\right)}dp\left(x\right)\\ \; & \to \int_{∥x∥\leq M}\frac{\left(\int e^{-\beta \rho (x,y)}\rho \left(x,y\right)dr^{*}\left(y\right)\right)}{\left(\int e^{-\beta \rho (x,y)}dr^{*}\left(y\right)\right)}dp\left(x\right),\mathrm{as}\;k\to \infty . \end{array}$$

Finally, combining the two parts, we have

$$\begin{array}{cc} \; & G_{q^{n_{k}}}\left(\beta \right)=\int \frac{\left(\int e^{-\beta \rho (x,y)}\rho \left(x,y\right)dq^{n_{k}}\left(y\right)\right)}{\left(\int e^{-\beta \rho (x,y)}dq^{n_{k}}\left(y\right)\right)}dp\left(x\right)-D\\ \; & \leq \int_{∥x∥\leq M}\frac{\left(\int e^{-\beta \rho (x,y)}\rho \left(x,y\right)dq^{n_{k}}\left(y\right)\right)}{\left(\int e^{-\beta \rho (x,y)}dq^{n_{k}}\left(y\right)\right)}dp\left(x\right)+\int_{∥x∥>M}\left(2A_{x}+1/\beta \right)dp\left(x\right)-D. \end{array}$$

Letting k→∞ first,

$$\begin{array}{cc} \; & \underset{k}{\lim\;\sup}G_{q^{n_{k}}}\left(\beta \right)\leq \;\;\int_{∥x∥\leq M}\;\;\frac{\left(\int e^{-\beta \rho (x,y)}\rho \left(x,y\right)dr^{*}\left(y\right)\right)}{\left(\int e^{-\beta \rho (x,y)}dr^{*}\left(y\right)\right)}dp\left(x\right)\\ \; & \;+\int_{∥x∥>M}\left(2A_{x}+1/\beta \right)dp\left(x\right)-D. \end{array}$$

Then letting M→∞,

$$\begin{array}{cc} \; & \underset{k}{\lim\;\sup}G_{q^{n_{k}}}\left(\beta \right)\leq \int \frac{\left(\int e^{-\beta \rho (x,y)}\rho \left(x,y\right)dr^{*}\left(y\right)\right)}{\left(\int e^{-\beta \rho (x,y)}dr^{*}\left(y\right)\right)}dp\left(x\right)-D=G_{r^{*}}\left(\beta \right), \end{array}$$

i.e., ${\lim\;\sup}_{k}G_{q^{n_{k}}}\left(\bar{\beta }+\varepsilon \right)\leq G_{r^{*}}\left(\bar{\beta }+\varepsilon \right)$. Meanwhile,

$$\begin{array}{cc} \; & G_{q^{n_{k}}}\left(\beta \right)=\int \frac{\left(\int e^{-\beta \rho (x,y)}\rho \left(x,y\right)dq^{n_{k}}\left(y\right)\right)}{\left(\int e^{-\beta \rho (x,y)}dq^{n_{k}}\left(y\right)\right)}dp\left(x\right)-D\\ \; & \geq \int_{∥x∥\leq M}\frac{\left(\int e^{-\beta \rho (x,y)}\rho \left(x,y\right)dq^{n_{k}}\left(y\right)\right)}{\left(\int e^{-\beta \rho (x,y)}dq^{n_{k}}\left(y\right)\right)}dp\left(x\right)-D. \end{array}$$

Letting k→∞ and then M→∞, we have

$$\begin{array}{cc} \; & \underset{k}{\mathrm{lim inf}}G_{q^{n_{k}}}\left(\beta \right)\geq \lim_{M\to \infty }\int_{∥x∥\leq M}\frac{\left(\int e^{-\beta \rho (x,y)}\rho \left(x,y\right)dr^{*}\left(y\right)\right)}{\left(\int e^{-\beta \rho (x,y)}dr^{*}\left(y\right)\right)}dp\left(x\right)-D=G_{r^{*}}\left(\beta \right), \end{array}$$

i.e., $\underset{k}{\mathrm{lim inf}}G_{q^{n_{k}}}\left(\bar{\beta }+\varepsilon \right)\geq G_{r^{*}}\left(\bar{\beta }+\varepsilon \right)$. Thus, we obtain $\lim_{k}G_{q^{n_{k}}}\left(\bar{\beta }+\varepsilon \right)=G_{r^{*}}\left(\bar{\beta }+\varepsilon \right)$. Similarly, $\lim_{k}G_{q^{n_{k}}}\left(\bar{\beta }-\varepsilon \right)=G_{r^{*}}\left(\bar{\beta }-\varepsilon \right)$. Using (A8),

$$\begin{array}{cc} G_{r^{*}}\left(\bar{\beta }+\varepsilon \right) & =\lim_{k}G_{q^{n_{k}}}\left(\bar{\beta }+\varepsilon \right)\leq \underset{k}{\mathrm{lim inf}}G_{q^{n_{k}}}\left({\tilde{\beta }}^{n_{k}}\right)\\ & \leq \underset{k}{\lim\;\sup}G_{q^{n_{k}}}\left({\tilde{\beta }}^{n_{k}}\right)\leq \lim_{k}G_{q^{n_{k}}}\left(\bar{\beta }-\varepsilon \right)=G_{r^{*}}\left(\bar{\beta }-\varepsilon \right). \end{array}$$

Then, let ε→0, $0=\lim_{k}G_{q^{n_{k}}}\left({\tilde{\beta }}^{n_{k}}\right)=G_{r^{*}}\left(\bar{\beta }\right)$. Therefore, $G_{r^{*}}\left(\bar{\beta }\right)=0=G_{r^{*}}\left(\beta^{*}\right)$. By the strict monotonicity, we have $\bar{\beta }=\beta^{*}$. Thus ${\tilde{\beta }}^{n}\to \beta^{*}$ holds. □

### Appendix A.4. Proof of Lemma 3

**Proof.** By [33] (Lemma 4.1), the distribution r of the reproduction variable is supported on ${\left[-M,M\right]}^{d}$ whenever ρ(x,y) is a strictly increasing, continuous difference distortion measure. We can just take the equidistant discretization nodes ${\left\{y_{j}^{n}\right\}}_{j=1}^{n}$ from ${\left[-M,M\right]}^{d}$. When $x,y\in {\left[-M,M\right]}^{d}$, exp(−βρ(x,y)) is a Lipschitz continuous function with respect to *y* with *L* being the Lipschitz constant. Then, we have the evaluation

$$\begin{array}{cc} \; & \left|\int \exp\left(-\beta \rho \left(x,y\right)\right)dq^{n}\left(y\right)-\int \exp\left(-\beta \rho \left(x,y\right)\right)dr^{*}\left(y\right)\right|\\ \; & \leq \sum_{i}\int_{I_{i}}|\exp\left(-\beta \rho \left(x,y\right)\right)-\exp\left(-\beta \rho \left(x,y_{i}^{n}\right)\right)|dr^{*}\left(y\right)\leq \sum_{i}\int_{I_{i}}L∥y-y_{i}^{n}∥dr^{*}\left(y\right)\leq Lh/2. \end{array}$$

We now establish the convergence rate:

$$\begin{array}{cc} f\left(q^{n}\right)-f\left(r^{*}\right) & =\int_{∥x∥\leq M}\;\;[\;\;-\;\log\;\left(\;\;\int \;\;\exp\left(-\beta \rho \left(x,y\right)\right)dq^{n}\left(y\right)\right)\\ \; & \;+\log\left(\int \exp\left(-\beta \rho \left(x,y\right)\right)dr^{*}\left(y\right)\right)]dp\left(x\right)\\ \; & =\int_{∥x∥\leq M}\log\frac{\int \exp\left(-\beta \rho \left(x,y\right)\right)dr^{*}\left(y\right)}{\int \exp\left(-\beta \rho \left(x,y\right)\right)dq^{n}\left(y\right)}dp\left(x\right)\\ \; & =\int_{∥x∥\leq M}\log\left(\frac{{\tilde{H}}_{r^{*}\left(y\right)}\left(x\right)-{\tilde{H}}_{q^{n}\left(y\right)}\left(x\right)}{\int \exp\left(-{\tilde{\beta }}^{n}\rho \left(x,y\right)\right)dq^{n}\left(y\right)}+1\right)dp\left(x\right)\\ \; & \leq \int_{∥x∥\leq M}\frac{\left|{\tilde{H}}_{r^{*}\left(y\right)}\left(x\right)-{\tilde{H}}_{q^{n}\left(y\right)}\left(x\right)\right|}{\int \exp\left(-{\tilde{\beta }}^{n}\rho \left(x,y\right)\right)dq^{n}\left(y\right)}dp\left(x\right)\\ \; & \leq \int_{∥x∥\leq M}\frac{\left|{\tilde{H}}_{r^{*}\left(y\right)}\left(x\right)-{\tilde{H}}_{q^{n}\left(y\right)}\left(x\right)\right|}{\delta_{0}}dp\left(x\right)\\ \; & \leq \int_{∥x∥\leq M}Lh/\left(2\delta \right)\;dp\left(x\right)\leq Lh/\left(2\delta \right)=O\left(h\right), \end{array}$$

where δ is the lower bound of $\int \exp\left(-\beta \rho \left(x,y\right)\right)dq^{n}\left(y\right)$ as shown in Theorem 1 and ${\tilde{H}}_{r(y)}\left(x\right)=\int \exp\left(-\beta \rho \left(x,y\right)\right)dr\left(y\right)$. Thus,

$$f\left(r^{n}\right)-f\left(r^{*}\right)=O\left(h\right).$$

Since the total number of discrete nodes is *n*, there are $n^{\frac{1}{d}}$ nodes in each dimension. So, the discretization step size is $\frac{2M}{n^{1/d}}$. Therefore, the convergence rate is $O(1/n^{\frac{1}{d}})$. □
